# Chemical Constituents from the Stems of *Ecdysanthera rosea*

**DOI:** 10.1007/s13659-014-0041-3

**Published:** 2014-11-02

**Authors:** Chang-Wei Song, Paul-Keilah Lunga, Xu-Jie Qin, Gui-Guang Cheng, Ya-Ping Liu, Xiao-Dong Luo

**Affiliations:** 1 State Key Laboratory of Phytochemistry and Plant Resources in West China, Kunming Institute of Botany, Chinese Academy of Sciences, Kunming, 650201 People’s Republic of China; 2 University of Chinese Academy of Sciences, Beijing, 100049 People’s Republic of China; 3 Laboratory of Phytobiochemistry and Medicinal Plants Study, Department of Biochemistry, Faculty of Science, University of Yaoundé 1, P.O. Box 812, Yaoundé, Cameroon

**Keywords:** *Ecdysanthera rosea*, Sesquiterpenoid, Phenolic glycoside, Absolute configuration

## Abstract

One new eudesmane sesquiterpenoid (**1**) named ecdysantherol A and two new benzene derivatives ecdysantherols B (**2)** and C (**3**), together with five known benzene derivatives (**4–8**) were isolated from the stems of *Ecdysanthera rosea*. The structures of the new compounds were elucidated by extensive spectroscopic methods and X-ray diffraction. The known compounds were identified by the comparison of their spectroscopic data with reported literature data. Compound **1** showed moderate antibacterial activity against the *Providensia smartii* with MIC value of 12.5 μg/mL.

**Electronic supplementary material:**

The online version of this article (doi:10.1007/s13659-014-0041-3) contains supplementary material, which is available to authorized users.

## Introduction

The *Ecdysanthera* comprises 15 species. Of which *Ecdysanthera rosea* is mainly distributed in tropical and subtropical areas of Asia and used as a traditional Chinese medicinal plant for the treatment of sore throat, chronic nephritis and trauma in China [1]. Terpenoids, benzene derivatives, steroids and their glycosides have been previously reported in this plant, they include three terpenoids and one steroid saponin with cytotoxic activities [2–12].

**Fig. 1:**
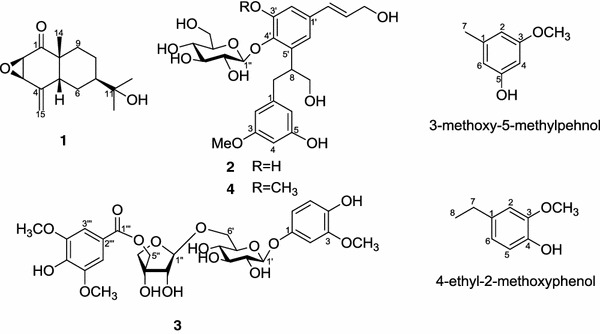
Chemical structures of compounds **1**–**4** and the standard chemicals

Given that the chemical constituents isolated from *E.rosea* are still limited and the existing bioactivity research of them are not related to its medicinal use directly. This attracted our attention to searching for more novel natural products from it. The present chemical investigation led to the isolation of three new compounds (**1–3**) (Fig. 1), and five known compounds: manglieside D (**4**) [13] erythro-guaiacylglycerol-*β*-*O*-4′-coniferyl alcohol (**5**) [14], (+)-(7*S*,8*R*)-guaiacylglycerol (**6**) [15], isocopoletin (**7**) [16], evofolin-B (**8**) [17] from this plant. In addition, preliminary test showed that compound **1** was a moderate antibacterial constituent against *Providensia smartii* with MIC value of 12.5 μg/mL, but a weak antibacterial constituent against *Enterococcus faecalis* and *Staphylococcus aureus* with MIC value of 50 μg/mL and 50 μg/mL respectively. In this paper, we report the isolation and structure elucidation of the new compounds.

## Results and Discussion

**Fig. 2:**
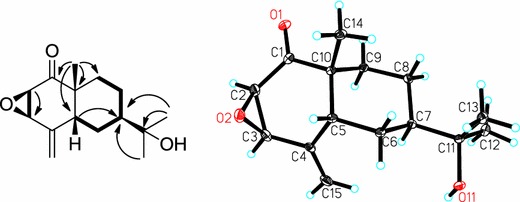
Selected ^1^H-^1^H COSY (), HMBC correlations (→) and the X-ray structure of **1**

The molecular formula of compound **1** was determined to be C_15_H_22_O_3_ by the HREIMS at *m/z* 250.1579 [M]^+^ (calcd. 250.1569), indicating five degrees of unsaturation. The ^13^C NMR and DEPT spectroscopic data exhibited 15 carbon signals for three methyls at *δ*_C_ 26.8 (q), 27.6 (q), 21.4 (q), four methylenes at *δ*_C_ 22.9 (t), 22.6 (t), 32.7 (t), 117.0 (t), four methines at *δ*_C_ 57.8 (d), 59.4 (d), 37.7 (d), 43.2 (d), and four quaternary carbons at *δ*_C_ 143.2 (s), 46.9 (s), 71.7 (s), *δ*_C_ 209.7 (s). Except for one ketone and a pair of double bond, it’s suggested that there should be a tricyclic structure in compound **1** to fit the three degrees of unsaturation. The ^1^H NMR spectrum displayed two olefinic protons at *δ*_H_ 5.68 (1H, d, *J* = 2.1 Hz, H-15a), 5.36 (1H, d, *J* = 2.1 Hz, H-15b), in accordance with a terminal double bond at *δ*_C_ 117.0 (t) in ^13^C NMR spectrum which was supported by the HSQC experiment. The protons at *δ*_H_ 3.42 (1H, d, *J* = 3.9 Hz, H-2) and *δ*_H_ 3.99 (1H, d, *J* = 3.9 Hz, H-3) with the same coupling constant indicated an epoxy moiety which was also supported by the ^1^H-^1^H COSY correlation between them (Fig. 2). The HMBC correlations from the singlet methyl signal at *δ*_H_ 1.21 (3H, s, H-14) to *δ*_C_ 209.7 (C-1), *δ*_C_ 37.7 (C-5), *δ*_C_ 32.7 (C-9), *δ*_C_ 46.9 (C-10); from the proton at *δ*_H_ 2.73 (H-5) to *δ*_C_ 143.2 (C-4), *δ*_C_ 117.0 (C-15), *δ*_C_ 43.2 (C-7), *δ*_C_ 22.9 (C-6) and from the terminal methyl signals at *δ*_H_ 1.15 (3H, s, H-12) and *δ*_H_ 1.15 (3H, s, H-13) to *δ*_C_ 71.7 (C-11) and *δ*_C_ 43.2 (C-7), together with the ^1^H-^1^H COSY correlations between *δ*_H_ 1.63 (H-6a) and *δ*_H_ 2.73 (H-5), *δ*_H_ 1.66 (H-7) proposed an eudesmane sesquiterpenoid skeleton of compound **1**. In addition, single-crystal X-ray diffraction (Fig. 2) using anomalous scattering of CuKα radiation (CCDC 1006467) revealed the absolute configuration of **1** as (2*R*,3*S*,5*S*,7*S*,10*S*)-2,3-epoxy-eudesm-4(15)-en-11-ol-1-one. Thus, compound **1** was elucidated as shown in Fig. 1, and named ecdysantherol A.

Compound **2** was isolated as a yellow powder. Its molecular formula C_25_H_32_O_11_ was deduced by the positive HR-ESIMS *m/z* 531.1841 [M + Na] ^+^. The NMR of **2** were very similar to the known compound manglieside D [13]. By comparison of the NMR data in literatures, the same structure segments of a 1,3,5-trisubstituted aromatic ring, a disubstituted *E*-configuration double bond and a sugar unit were confirmed [18, 19]. The major difference was that compound **2** possessed a different 1′,3′,4′, 5′-tetrasubstituted aromatic ring, in which a methoxy group at C-3*′* of manglieside D was replaced by a hydroxy group and this could be confirmed by its molecular formula and different proton signals at *δ*_H_ 6.81 (1H, d, *J* = 1.8 Hz, H-2′), *δ*_H_ 6.83 (1H, d, *J* = 1.8 Hz, H-6′). Therefore, compound **2** was elucidated as shown in Fig. 1, and named ecdysantherol B Fig. 1.

Confusingly, one literature neglected coupling constant and assigned same coupling pattern as 3-OCH_3_ and 4-OH substituted aromatic ring, because NOE correlation of methoxyl proton with only one aromatic proton (C-2) was observed in ROESY spectrum, which deduced a substituted aromatic carbon (C-4) [20]. Interestingly, same NOE correlation pattern was observed in ROESY spectrum of **2**. Then, ^1^H NMR spectral data of compound **2** were further collected in different solvent, and the result indicated same coupling pattern without large coupling constant to meet *Ortho*-proton in aromatic ring. To further confirmed our assignment, we ordered standard chemicals of 3-methoxy-5-methylpehnol (CAS NO. 3209-13-0), and 4-ethyl-2-methoxyphenol (CAS NO. 2785-89-9), and ^1^H NMR spectral data of two compounds were record in DMSO-*d6*. The same coupling pattern of compound **2** and 3-methoxy-5-methylpehnol unambiguously confirmed 1,3,5-trisubstituted aromatic ring and proposed that no observation of NOE correlation between –OCH_3_ with both aromatic protons (H-2 and H-4) was not sufficient reason for 1,3,4-trisubstituted assignment. Besides, chemical shift of –OH in DMSO-*d6* might be a characteristic for 3-methoxy-4-hydroxy substituted (ca *δ*_H_ 8.7) and 3-methoxy-5-hydroxy substituted (ca *δ*_H_ 9.3) in aromatic ring.

Compound **3** was isolated as a yellow powder. The molecular formula C_27_H_34_O_16_ was deduced by HRESIMS at *m/z* 614 [M + Na]^+^. Detailed analysis of NMR data indicated that **3** had a similar structure to that of the known compound previously reported, [21] except for the substituent groups on the aromatic ring. By comparison of its NMR data with those reported in literature, the signals at *δ*_H_ 7.34 (1H, s, H-3′′′), 7.34 (1H, s, H-7′′′) showed that **3** had a different 1,3,4,5-tetra-substituted aromatic ring. In addition, the HMBC correlations from *δ*_H_ 3.77 (3H, s, -OMe) to 149.1 (C-3) suggested it had another different 1,2,4-tri-substituted aromatic ring with the known compound. The above observations indicated that compound **3** was an analogue of the known compound. Furthermore, the detailed 2D NMR spectroscopic data revealed the position of the hydroxy groups and methoxy groups in compound **3**. Thus, compound **3** was elucidated as shown in Fig. 1, and named ecdysantherol C.

## Experimental Section

### General Experimental Procedures

Optical rotations were obtained with a Jasco P-1020 Automatic Digital Polariscope. UV spectrum was measured with a Shimadzu UV2401PC in MeOH solution. IR spectra (KBr) were obtained on a Bruker tensor-27 infrared spectrophotometer. ^1^H, ^13^C, and 2D NMR spectra were recorded on a Bruker AM-400, a DRX-500 NMR and an Avance III 600 spectrometer with TMS as internal standard. MS data were obtained on a Waters Autospec Premier P776 for HREI. An APEX DUO (Bruker) instrument was used for the single crystal X-ray diffraction. Column chromatography (CC) was performed on Silica gel (200–300 mesh, Qingdao Marine Chemical Ltd., Qingdao, People’s Republic of China) and RP-18 gel (20–45 *µ*m, Fuji Silysia Chemical Ltd., Tokyo, Japan). Fractions were monitored by TLC (GF 254, Qingdao Haiyang Chemical Co., Ltd., Qingdao, People’s Republic of China), and spots were visualized by 10 % H_2_SO_4_-ethanol reagent.

### Plant Material

The dried stems of *E. rosea* were collected from Xishuangbanna Autonomous Prefecture, Yunnan Province, People’s Republic of China, and identified by Jingyun Cui of Xishuangbanna Botanic Garden. A voucher specimen (Cui 200811-03) has been deposited at the Herbarium of Kunming Institute of Botany, Chinese Academy of Sciences.

### Extraction and Isolation

The air-dried and smashed stems of *E. rosea* (10 kg) were extracted with MeOH three times at room temperature. After in vacuum pump evaporation of the solvent, the combined crude extract was suspended in H_2_O and extracted with ethyl acetate three times. The EtOAc fraction (129.0 g) was eluted with gradient mixtures of CHCl_3_–MeOH (100:1 → 1:1) on silica gel column to yield 5 fractions [Fr.A (38.5 g), Fr.B (13 g), Fr.C (14 g), Fr.D (9 g), Fr.E (18 g)]. Fraction B (13 g) was isolated by Sephadex LH-20 and repeated silica gel column to yield compound **1** (11 mg). Fraction D (9 g) was chromatographed over Sephadex LH-20 (MeOH), MPLC (MeOH–H_2_O) and HPLC (MeCN–H_2_O) to provide compound **5** (7 mg). Fraction E (18 g) was subjected to the Sephadex LH-20, eluted with MeOH–H2O (1:1) and chromatographed over RP-C_18_ gel (MeOH–H2O ) to afford compounds **2** (6 mg), **3** (5 mg), **4** (17 mg), **6** (14 mg), **7** (14 mg) and **8** (8 mg).

Compound **1**; colorless needle crystal; [*α*]_D_^21.5^ + 67.0 (*c* 0.1, MeOH); IR (KBr) *ν*_max_ 3360, 1635 cm^−1^; ^1^H (400 MHz) and ^13^C NMR (100 MHz) data (MeOH), see Table 1, HREIMS *m/z* 250.1579 (calcd for C_15_H_22_O_3_, 250.1569).

**Table 1:** NMR Data of **1**–**3** (*δ* in ppm and *J* in Hz)

No.	1^a^		No.	2^b^		No.	3^b^	
	*δ*_H_	*δ*_C_		*δ*_H_	*δ*_C_		*δ*_H_	*δ*_C_
1		209.7 s	1		133.1 s	1		152.7 s
2	3.42 (d, 3.9)	57.8 d	2	6.68 (br s)	114.1 d	2	6.69 (d, 2.6)	103.8 d
3	3.99 (d, 3.9)	59.4 d	3		148.4 s	3		149.1 s
4		143.2 s	4	6.63 (br s)	115.7 d	4		142.9 s
5	2.73 (br s)	37.7 d	5		145.4 s	5	6.65 (d, 8.6)	116.0 d
6	2.13 (m) 1.63 (m)	22.9 t	6	6.63 (br s)	122.7 d	6	6.54 (dd, 8.6, 2.6)	109.8 d
7	1.66 (m)	43.2 d	7	2.90 (m)	38.1 t	1′	4.68 (d, 7.3)	103.6 d
8	1.64 (m) 1.34 (m)	22.6 t	8	4.01 (m)	42.3 d	2′	3.40 (m)	74.9 d
9	1.37 (m); 1.33 (m)	32.7 t	9	3.69 (dd, 10.4, 6.9) 3.63 (dd, 10.4, 6.3)	66.6 t	3′	3.41 (m)	77.9 d
10		46.9 s	1′		135.6 s	4′	3.33 (m)	71.5 d
11		71.7 s	2′	6.81 (d, 1.8)	113.2 d	5′	3.51 (m)	76.7 d
12	1.15 (s)	26.8 q	3′		150.9 s	6′	4.03 (d, 11.0) 3.62 (dd, 11.0, 6.2)	68.4 t
13	1.15 (s)	27.6 q	4′		144.9 s	1″	5.02 (d, 2.1)	110.5 d
14	1.21 (s)	21.4 q	5′		138.1 s	2″	3.98 (d, 2.1)	78.7 d
15	5.68 (d, 2.1) 5.36 (d, 2.1)	117.0 t	6′	6.83 (d, 1.8)	118.5 d	3″		79.0 s
			7′	6.49 (d, 15.8)	131.4 d	4″	4.08 (d, 9.8) 3.86 (d, 9.8)	75.0 d
			8′	6.23 (dt, 15.8, 5.6)	129.4 d	5″	4.40 (d, 11.3) 4.33 (d, 11.3)	67.8 t
			9′	4.19 (dd, 5.5, 1.2)	63.6 t	1′′′		167.8 s
			1**″**	4.57 (d, 7.8)	107.0 d	2′′′		121.0 s
			2″	3.50 (m)	75.5 d	3′′′	7.34 (s)	108.3 d
			3″	3.38 (m)	77.9 d	4′′′		148.8 s
			4″	3.45 (m)	70.7 d	5′′′		141.9 s
			5″	3.19 (m)	78.3 d	6′′′		148.8 s
			6″	3.78 (m,) 3.73 (m)	61.9 t	7′′′	7.34 (s)	108.3 d
			-OMe	3.73 (s)	56.3 q	-OMe	3.77 (s)	56.3 q
						-OMe	3.86 (s)	56.8 q
						-OMe	3.86 (s)	56.8 q

^a^ Measured in chloroform-*d*_3_

^b^ Measured in methanol-*d*_4_

Compound **2**; yellow, amorphous powder; [*α*]_D_^21.3^ + 28.3 (*c* 0.1, MeOH); UV (MeOH) *λ*_max_ (log *ε*) 245.6 (4.12), 203.0 (4.65) nm; IR (KBr) *ν*_max_ 3425, 2985, 1029 cm^−1^; ^1^H (400 MHz) and ^13^C NMR (100 MHz) data, see Table 1, HREIMS *m/z* 531.1851 (calcd for C_25_H_32_O_11_Na, 531.1842).

Compound **3**; yellow, amorphous powder; [*α*]_D_^22.3^ – 64.1 (*c* 0.15, MeOH); UV (MeOH) *λ*_max_ (log *ε*) 284.2 (4.05), 202.8 (4.57) nm; IR (KBr) *ν*_max_ 3431, 1049 cm^−1^; ^1^H (400 MHz) and ^13^C NMR (100 MHz) data, see Table 1, HREIMS *m/z* 637.1754 (calcd for C_27_H_34_O_16_Na, 637.1745).

### Antimicrobial assays

The microorganisms used in the antimicrobial assay were obtained from the American Type Culture Collection (ATCC). They included three bacteria strains: *E. faecalis* ATCC 10541, *S. aureus* ATCC 25922 and *Providensia smartii* ATCC29916. The MIC values of the compounds were determined by the broth microdilution method in 96-well microtitre. The 96-well plates were prepared by dispensing into each well 100 μL of Mueller–Hinton broth for bacteria. The test substances were initially prepared in 10 % DMSO in broth medium at 400 μg/mL for compounds or 50 *μ*g/mL for the reference antibiotics, gentamycin. A volume of 100 μL of each test sample was added into the first wells of the microtitre plate (whose wells were previously loaded with 100 μL of broth medium). Serial two-fold dilutions of the test samples were made and 100 μL of bacterial inoculum standardized at 10^6^ CFU/mL were added. This gave final concentration ranges from 100 to 0.781 μg/mL for the compounds and 12.5 to 0.097 μg/mL for reference substance. The plates were sealed with parafilm, then agitated with a plate shaker to mix their contents and incubated at 35 °C for 24 h.

MICs were determined upon addition of 50 μL (0.2 mg/mL) *p*-iodonitrotetrazolium chloride (INT, Sigma-Aldrich, South Africa). Viable bacteria reduced the yellow dye to a pink color. The MIC corresponded to the lowest well concentration where no color turbidity change was observed, indicating no growth of microorganism. All tests were performed in triplicates.

### Crystallographic Data of **1**

C_15_H_22_O_3_, *M* = 250.33, orthorhombic, *a* = 5.85670(10) Å, *b* = 11.1899(2) Å, *c* = 19.6555(3) Å, *α* = 90.00°, *β* = 90.00°, *γ* = 90.00°, *V* = 1288.14(4) Å^3^, *T* = 100(2) K, space group *P*212121, *Z* = 4, *μ*(CuKα) = 0.706 mm^−1^, 7204 reflections measured, 2162 independent reflections (*R*_*int*_ = 0.0345). The final *R*_*1*_ values were 0.0346 (*I* > 2*σ*(*I*)). The final *wR*(*F*^2^) values were 0.1028 (*I* > 2*σ*(*I*)). The final *R*_*1*_ values were 0.0347 (all data). The final *wR*(*F*^2^) values were 0.1029 (all data). The goodness of fit on *F*^2^ was 1.130. Flack parameter = 0.1(2). The Hooft parameter is 0.06(6) for 852 Bijvoet pairs. The crystal structure of compound 1 was solved by direct method SHELXS-97 and expanded using the difference Fourier techniques, refined by the program SHLXL-97 and the full-matrix least-squares calculations. Crystallographic data for the structure of compound **1** have been deposited with the Cambridge Crystallographic data centre (deposition no. CCDC 1006467). Copies of these data can be obtained free of charge via www.ccdc.cam.ac.uk.

## Electronic supplementary material

Below is the link to the electronic supplementary material.

(DOC 5207 kb)
