# Supplementary material for: Chemical Constituents from the Stems of Ecdysanthera rosea
Source: Nat Prod Bioprospect. 2014 Nov 2;4(6):319–23. doi: 10.1007/s13659-014-0041-3 (PMC4250565; doi:10.1007/s13659-014-0041-3)
Supplement: Supplementary file 1 — (DOC 5207 kb) [file 13659_2014_41_MOESM1_ESM.docx]

Electronic Supplementary Material

**Chemical constitutes from the stems of *Ecdysanthera rosea***

Chang-Wei Song ^a, b^, Paul-Keilah Lunga ^a,c^, Xu-Jie Qin^a, b^, Gui-Guang Cheng^a, b^, Ya-Ping Liu ^a,^* , and Xiao-Dong Luo ^a,^*

^a^ State Key Laboratory of Phytochemistry and Plant Resources in West China, Kunming Institute of Botany, Chinese Academy of Sciences, Kunming 650201, People’s Republic of China

^b^ University of Chinese Academy of Sciences, Beijing 100049, People’s Republic of China

^c^ *Department of Biochemistry, Laboratory of Phytobiochemistry and Medicinal Plants Study, Faculty of Science, University of Yaoundé 1, Yaoundé P.O. Box 812, Cameroon*

**Figure 1.** The structures of compounds **1**‒**3**

Figures 1-8 NMR, MS spectra of **1**.

Figures 9-17 NMR, MS spectra of **2**.

Figures 18-25 NMR, MS spectra of **3**.

Figure 26. ^1^H NMR spectrum of 3-methoxy-5-methylpehnol (600 MHz, dmso-*d*_6_).

Figure 27. ^1^H NMR spectrum of 4-ethyl-2-methoxyphenol (600 MHz, dmso-*d*_6_).

**
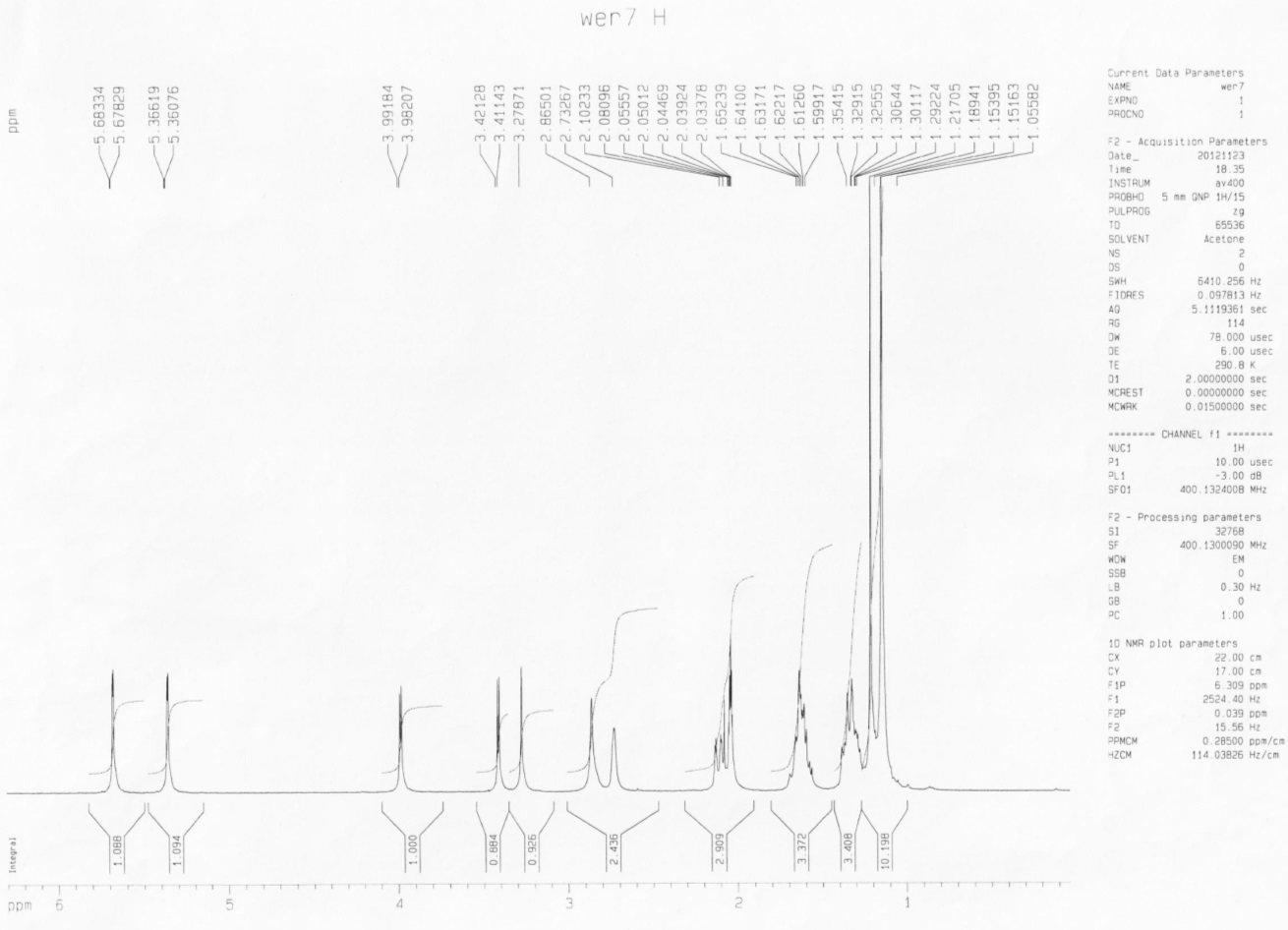
**

**Figure 1.** ^1^H NMR spectrum of **1** (400 MHz, acetone-*d*_6_).**
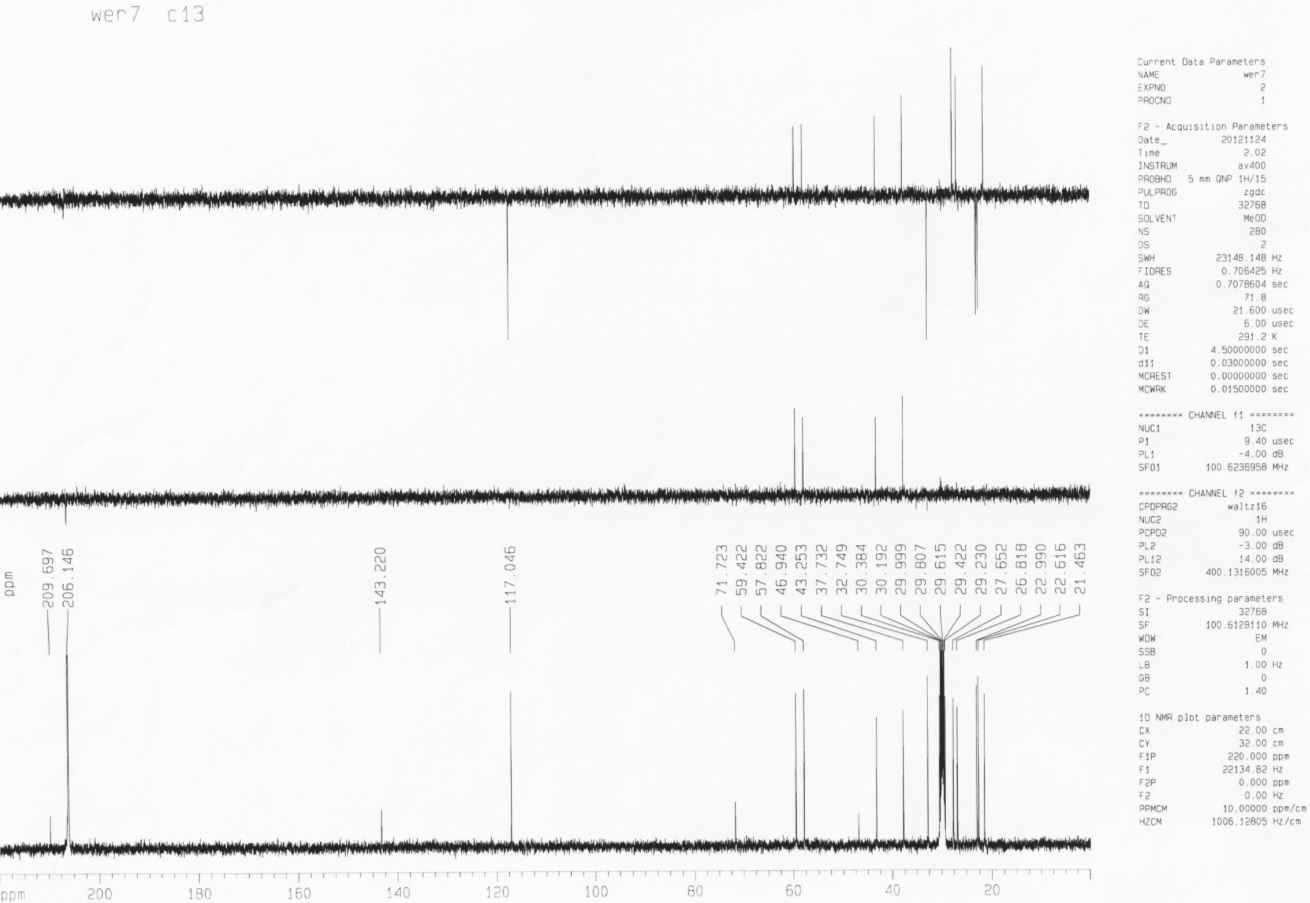
**

**Figure 2.** ^13^C NMR spectrum of **1** (400 MHz, acetone-*d*_6_).
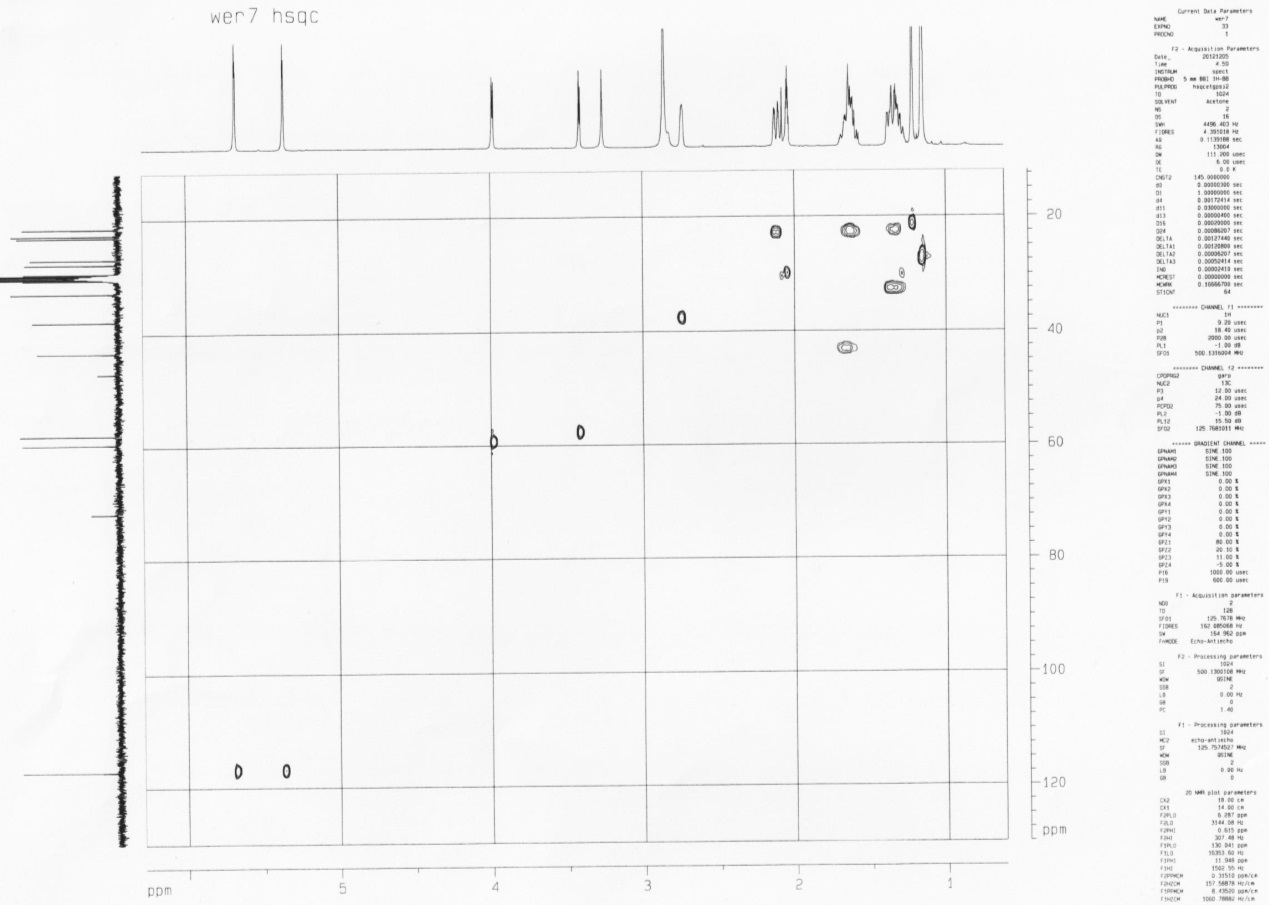


**Figure 3.** HSQC spectrum of **1** (400 MHz, acetone-*d*_6_).

**
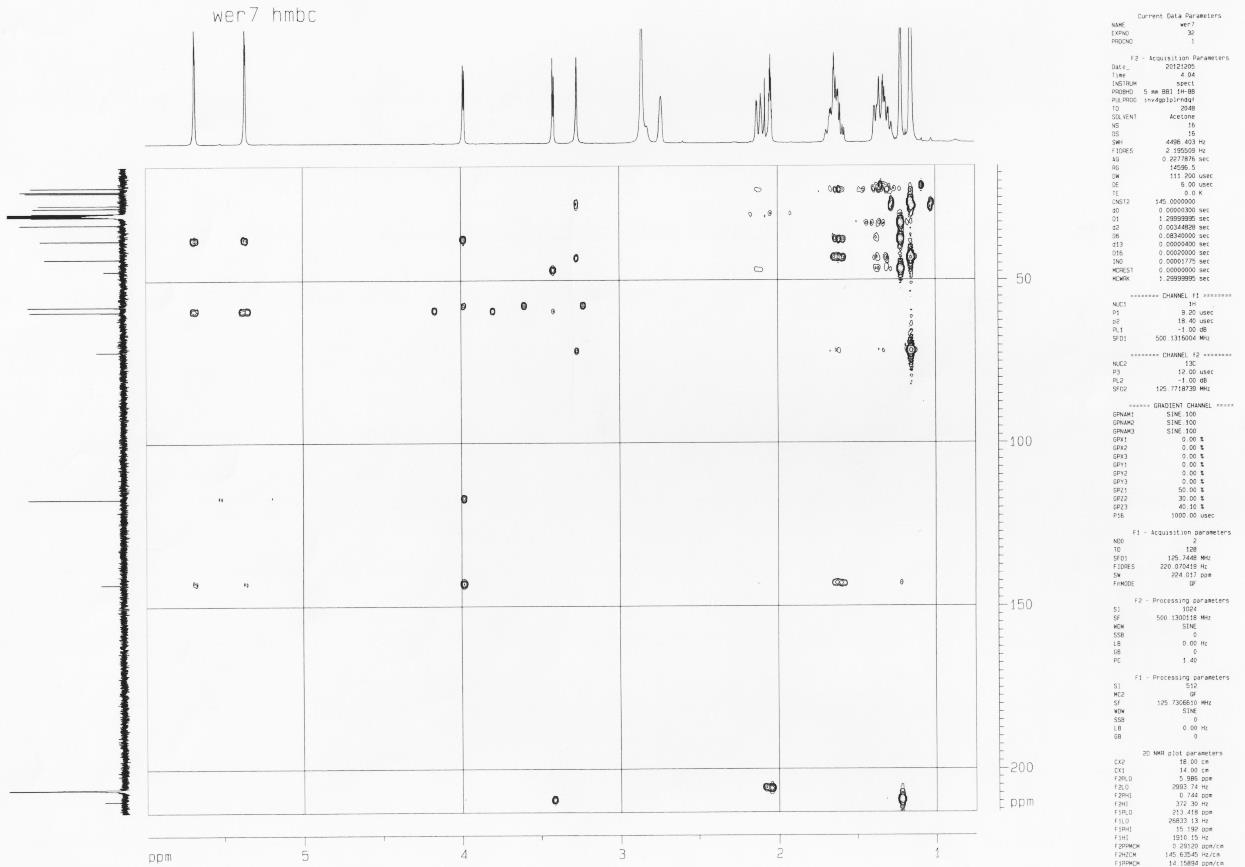
**

**Figure 4.** HMBC spectrum of **1** (400 MHz, acetone-*d*_6_).
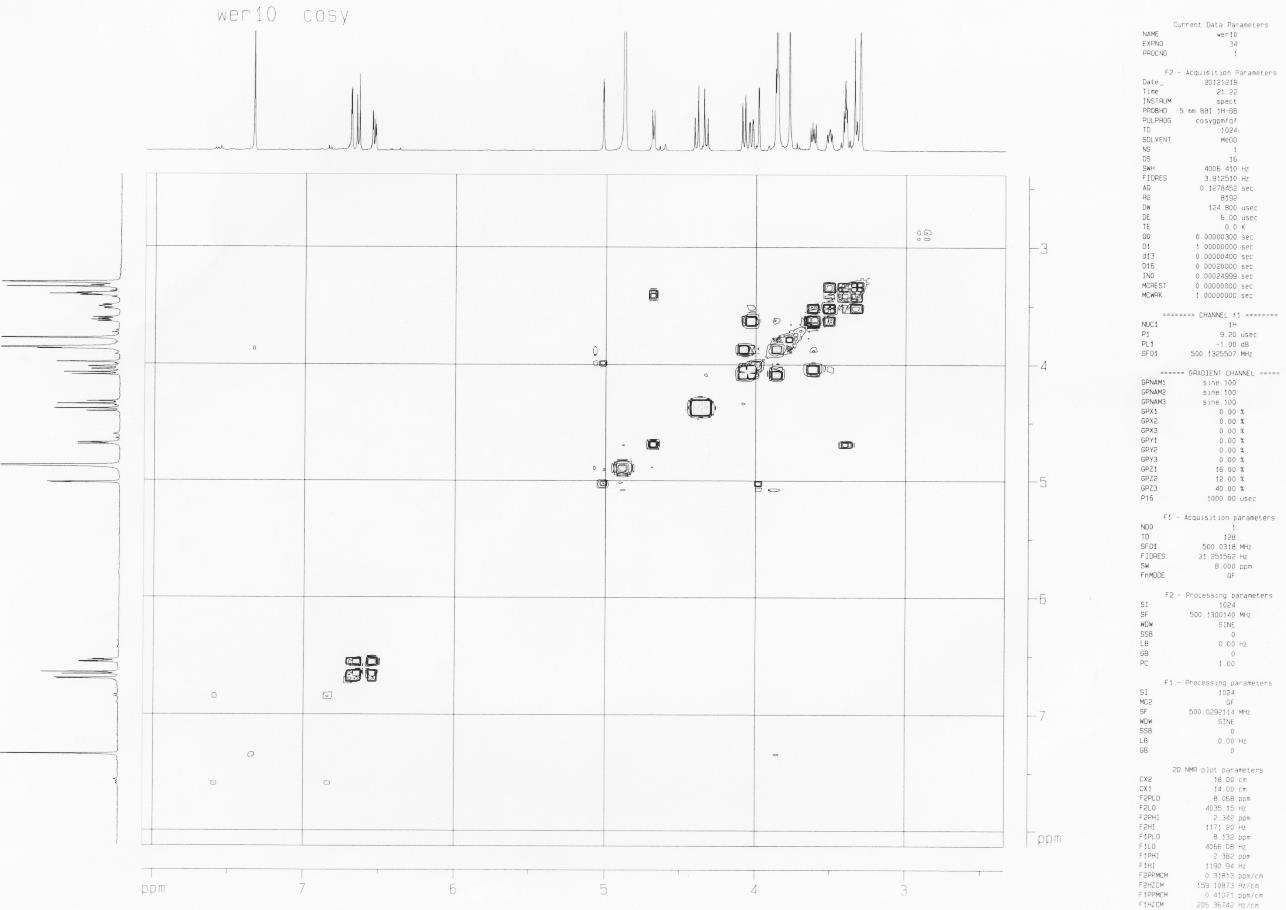


**Figure 5.** COSY spectrum of **1** (400 MHz, acetone-*d*_6_).

**
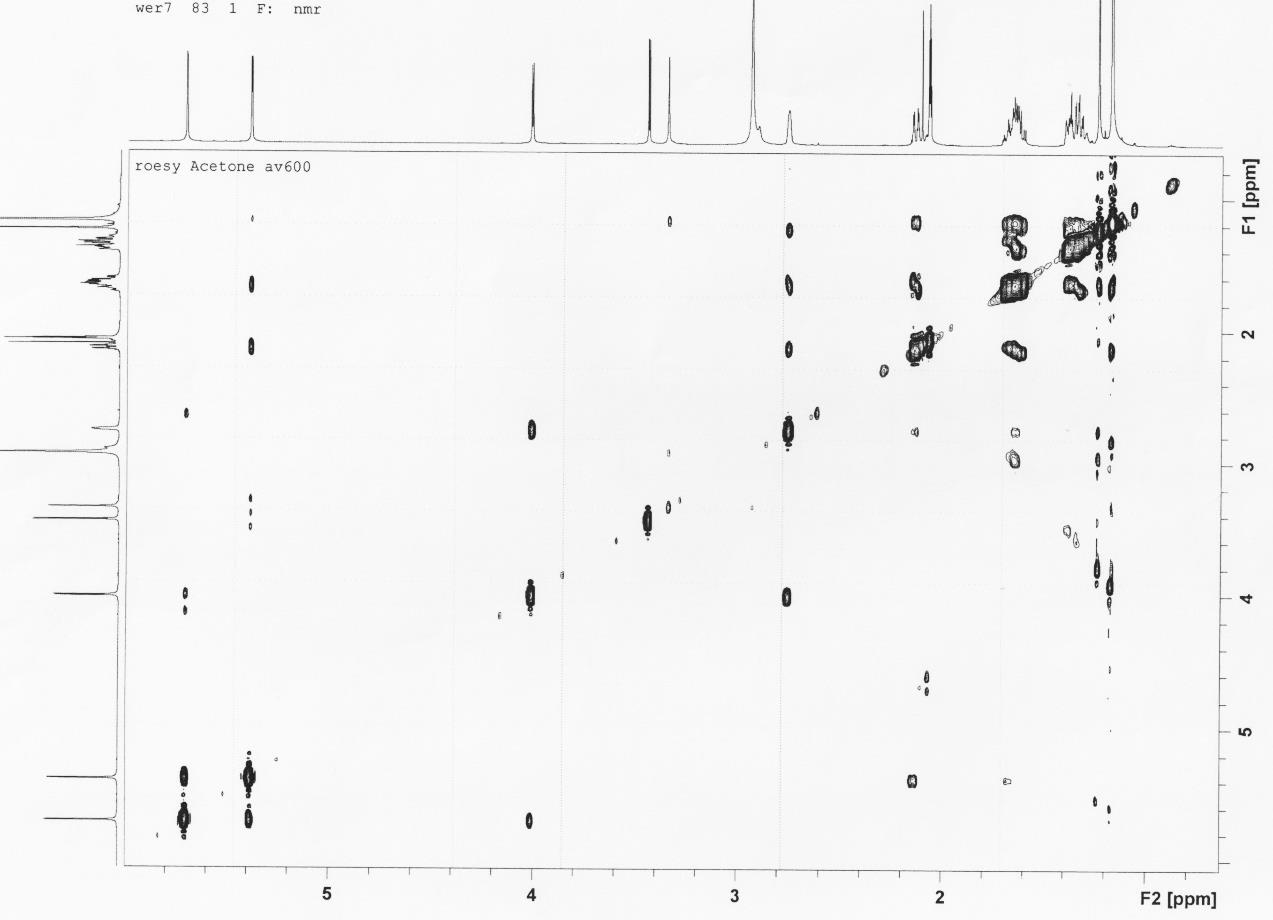
**

**Figure 6.** ^1^H NMR spectrum of **1** (400 MHz, acetone-*d*_6_).


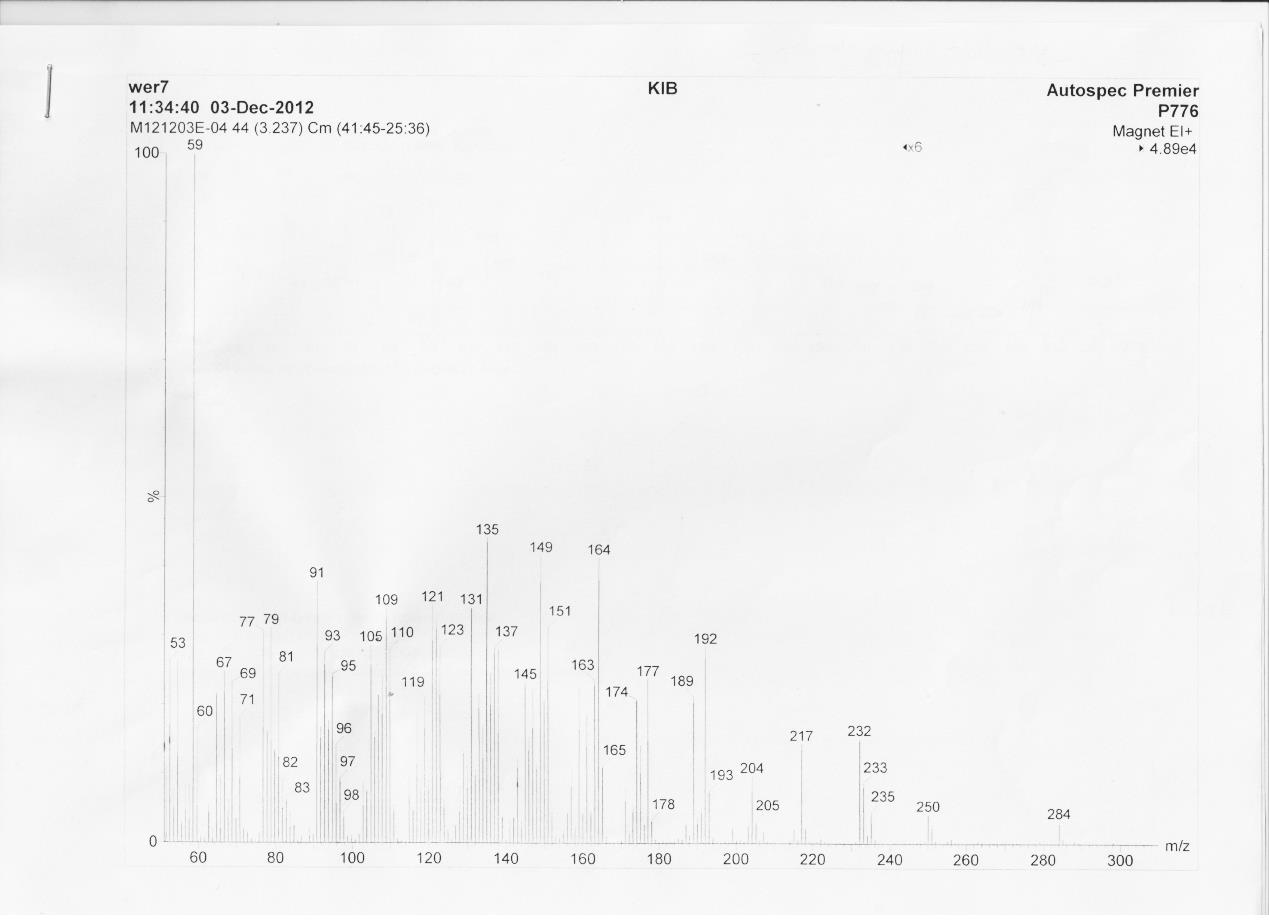


**Figure 7.** EI-MS spectrum of **1**


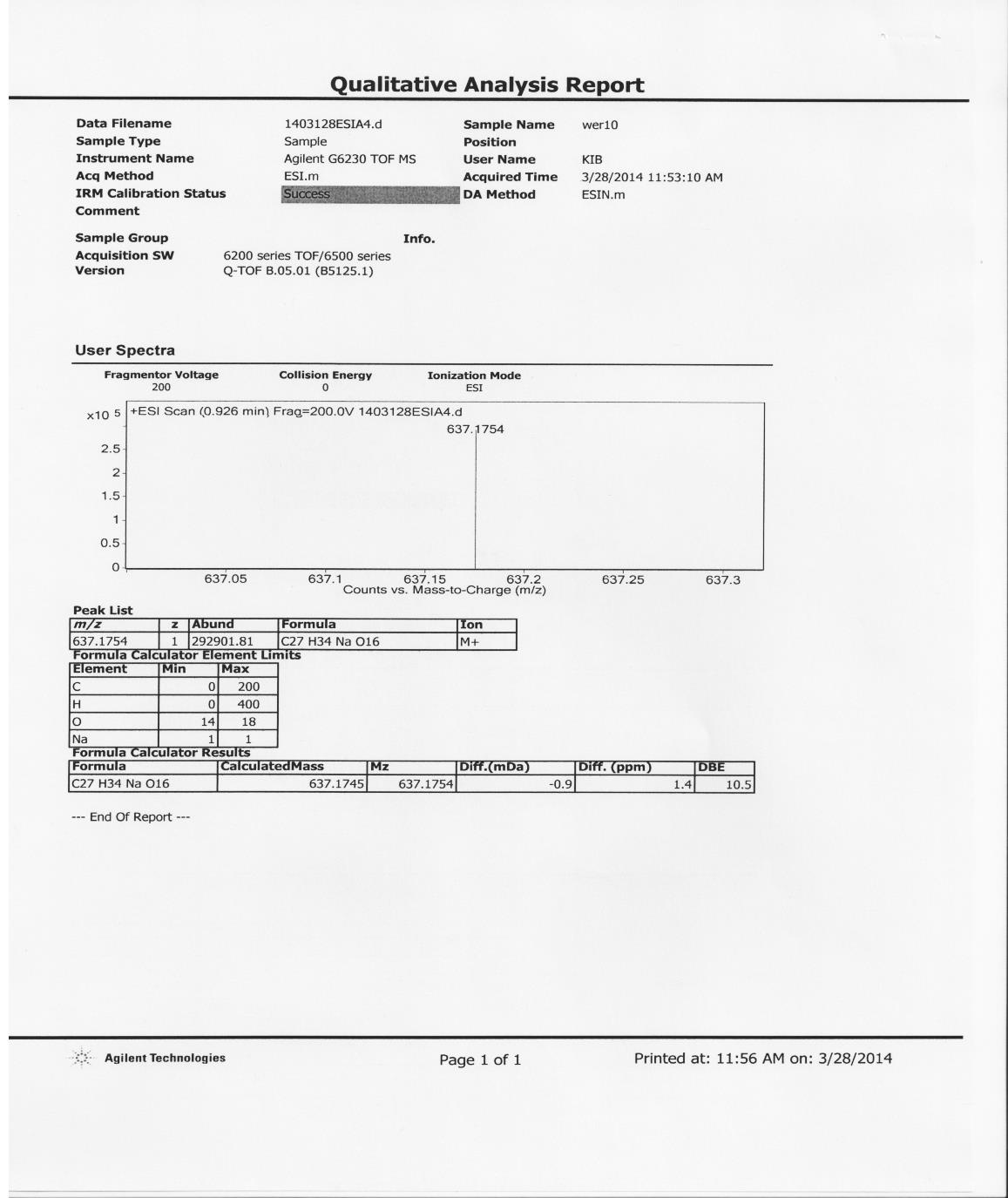


**Figure 8. HR**EI-MS spectrum of **1**


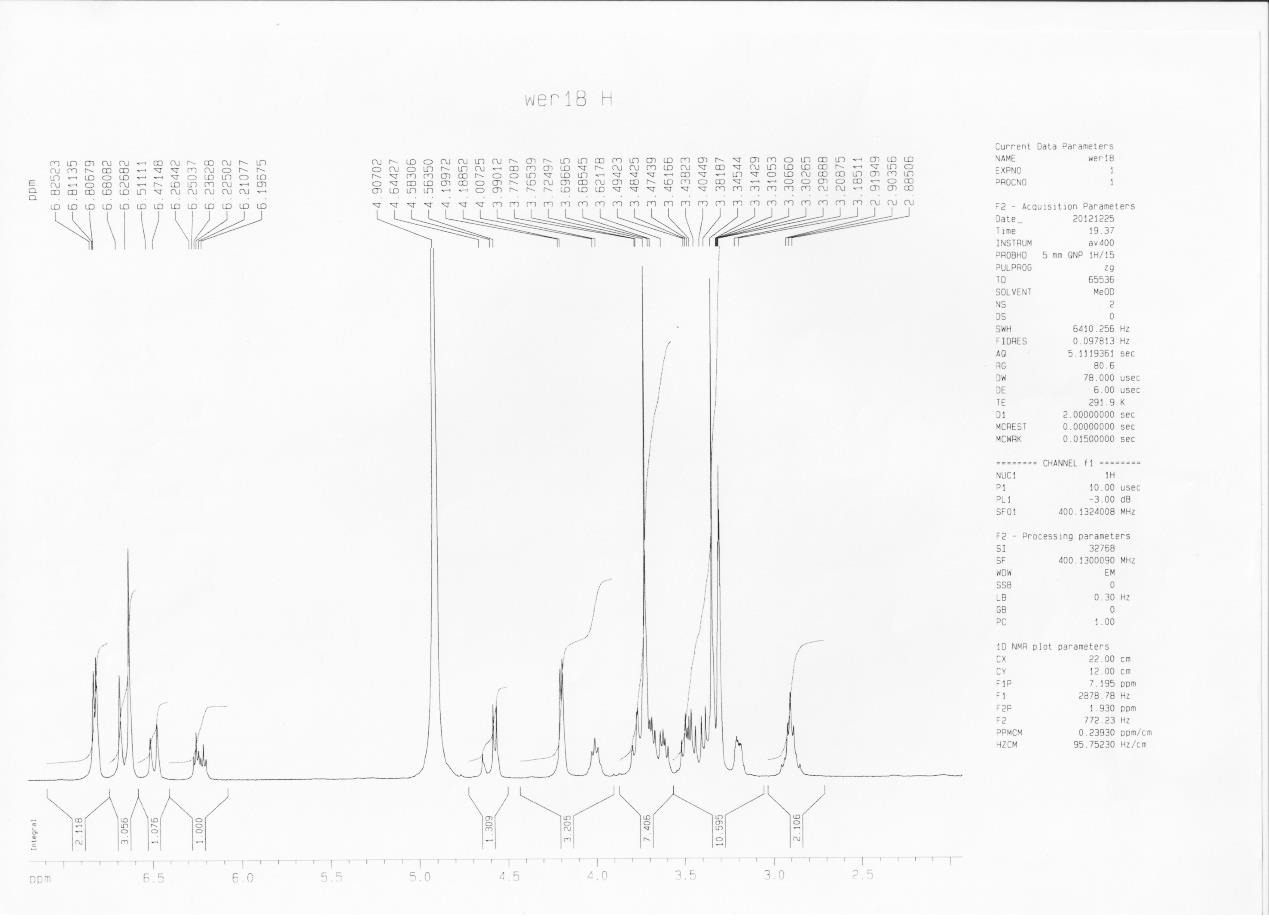


**Figure 9.** ^1^H NMR spectrum of **2** (400 MHz, methanol-*d*_4_).


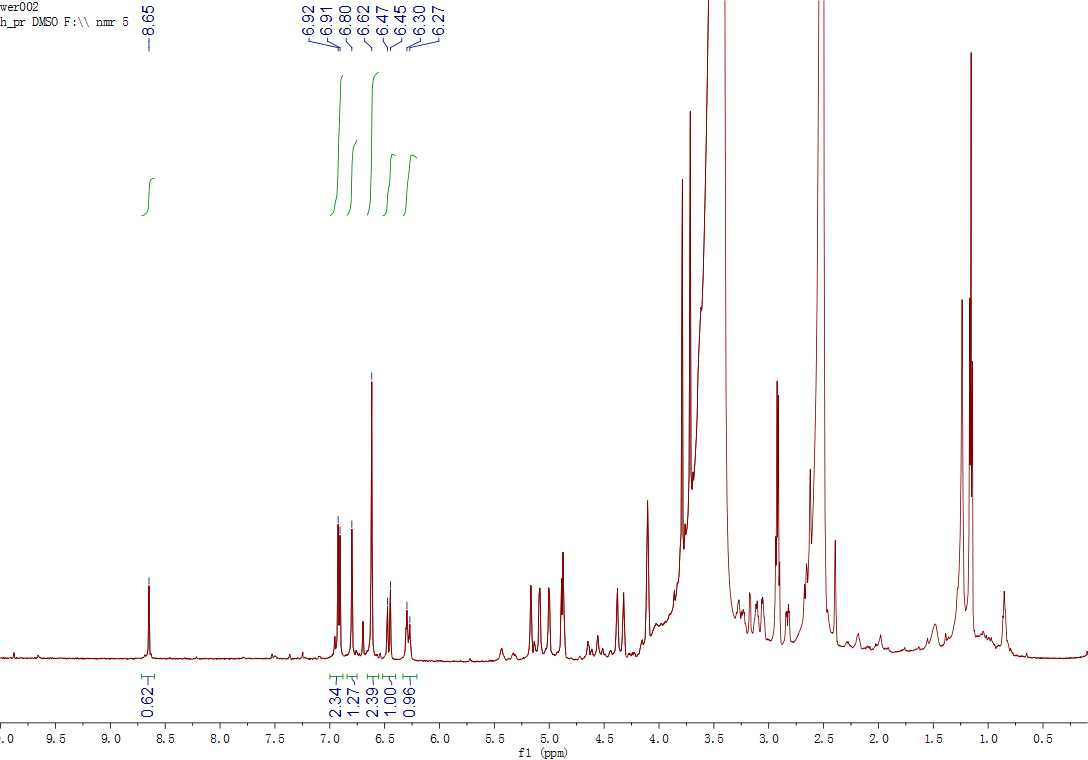


**Figure 10.** ^1^H NMR spectrum of **2** (600 MHz, dmso-*d*_6_).


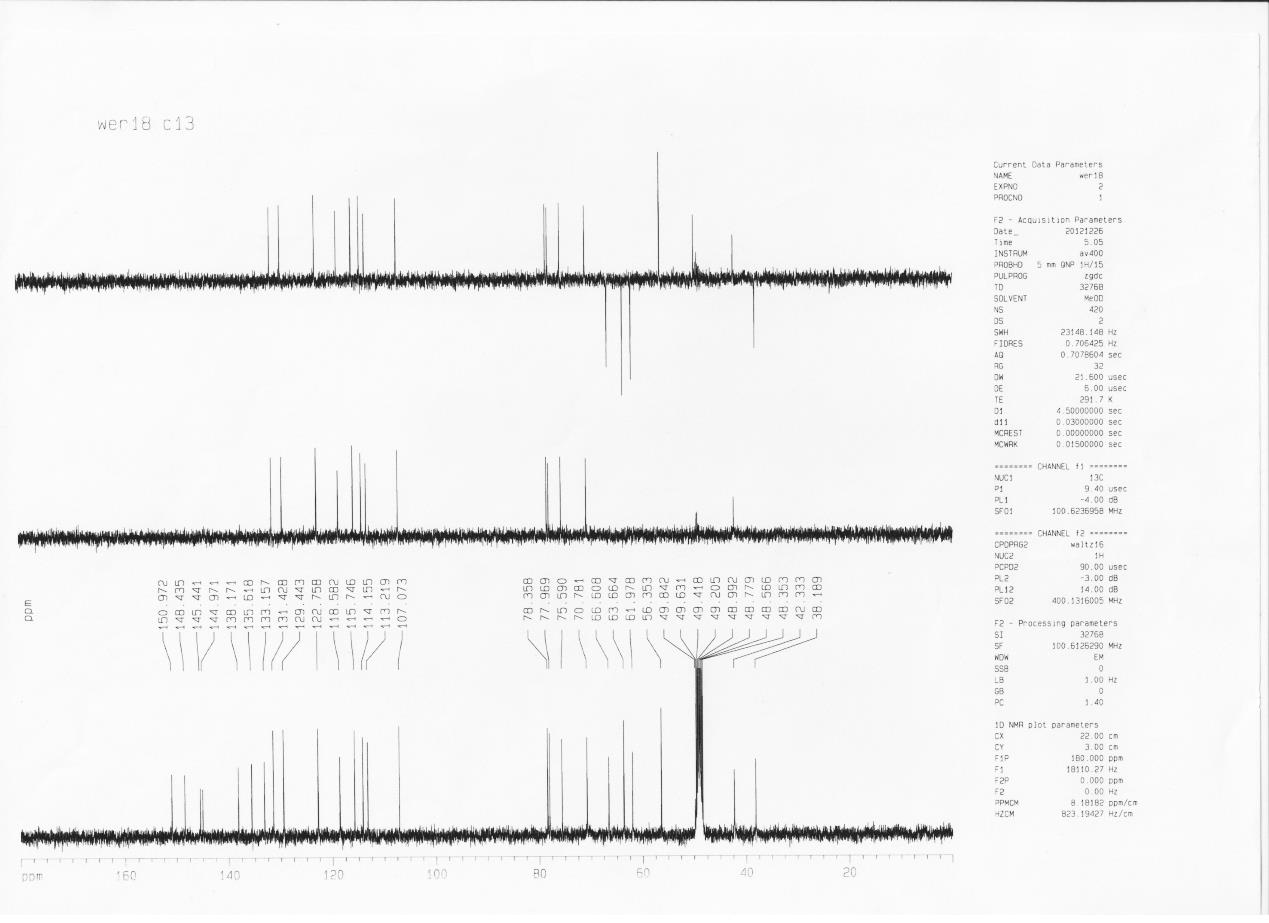


**Figure 11.** ^13^C NMR spectrum of **2** (400 MHz, methanol-*d*_4_).


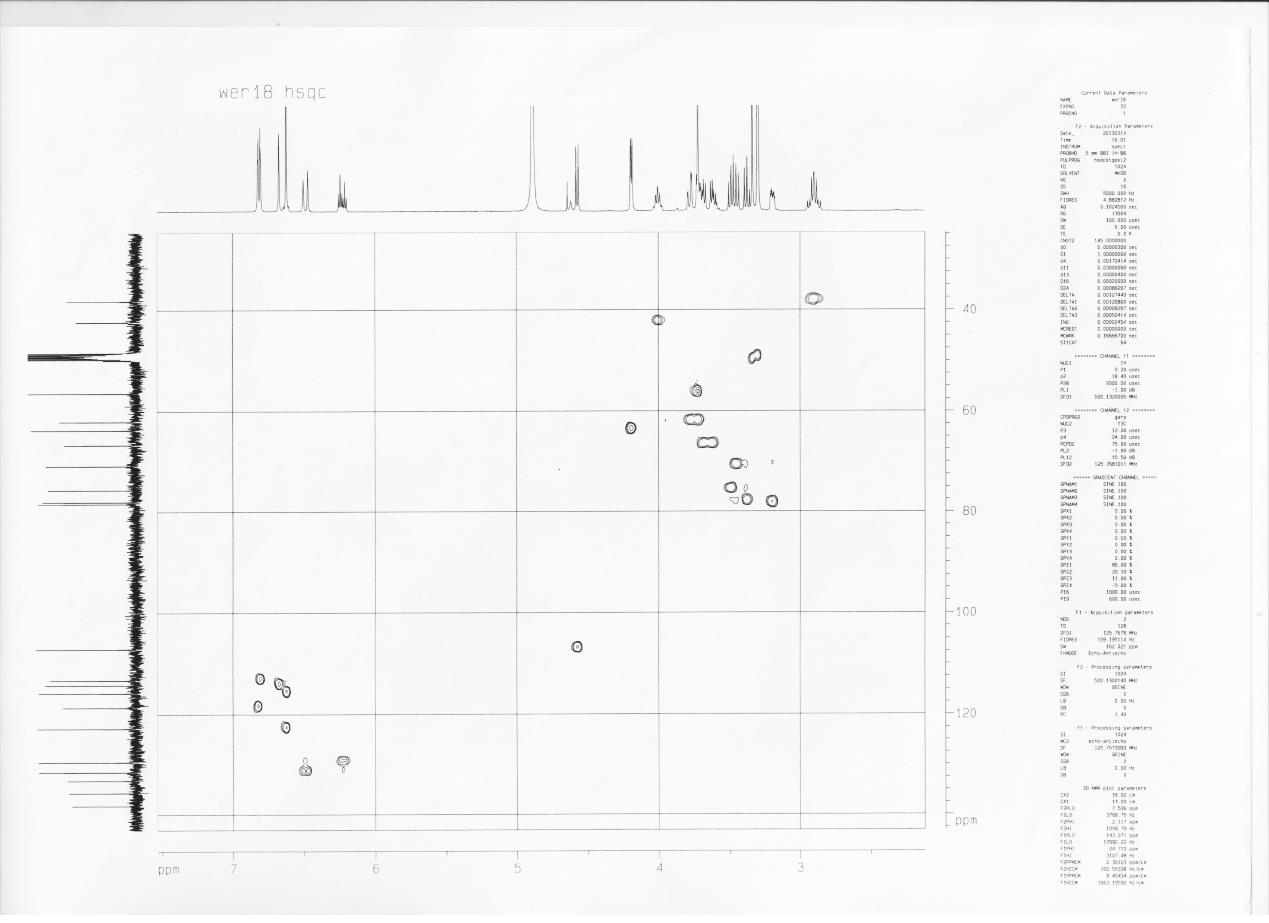


**Figure 12.** HSQC spectrum of **2** (400 MHz, methanol-*d*_4_).**
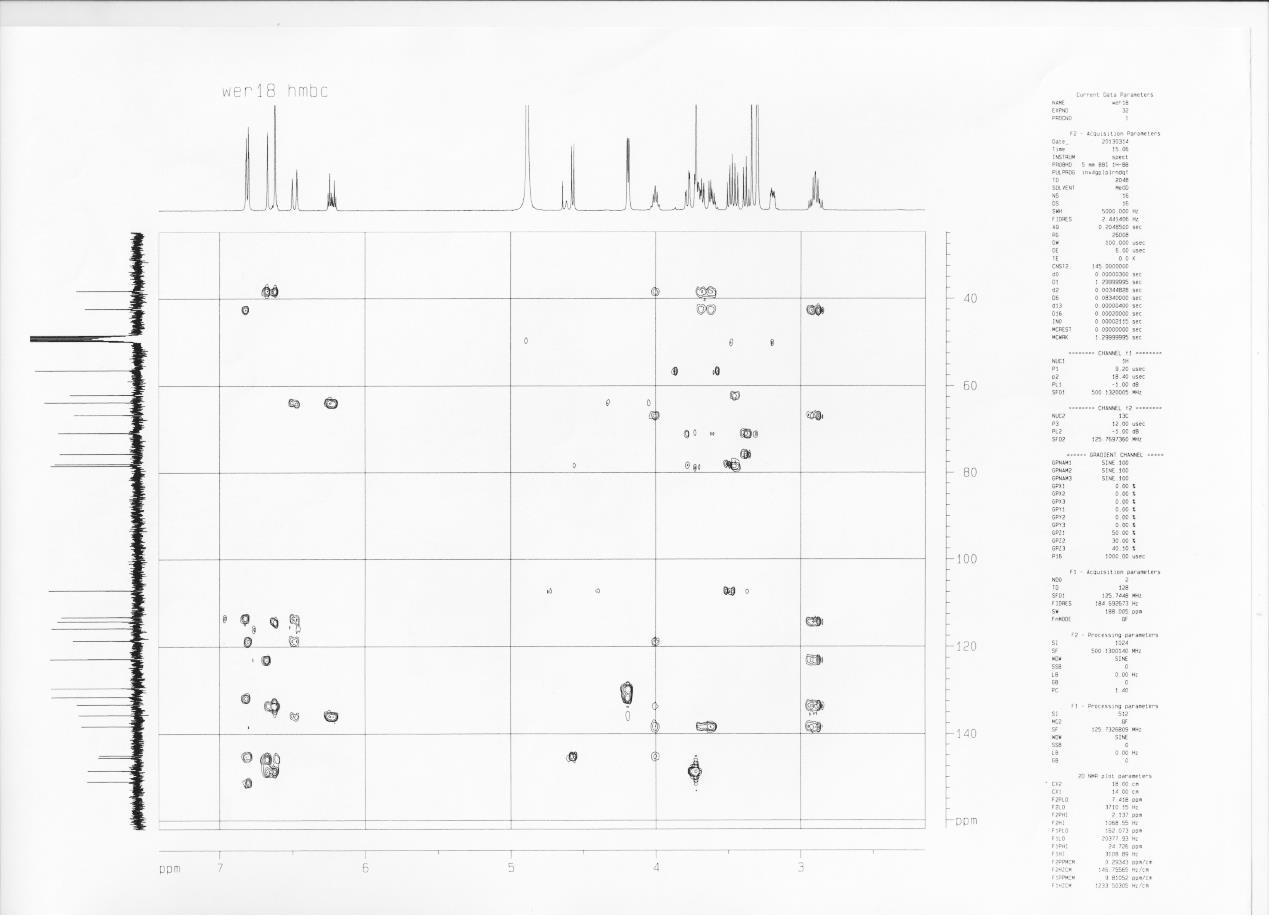
**

**Figure 13.** HMBC spectrum of **2** (400 MHz, methanol-*d*_4_).
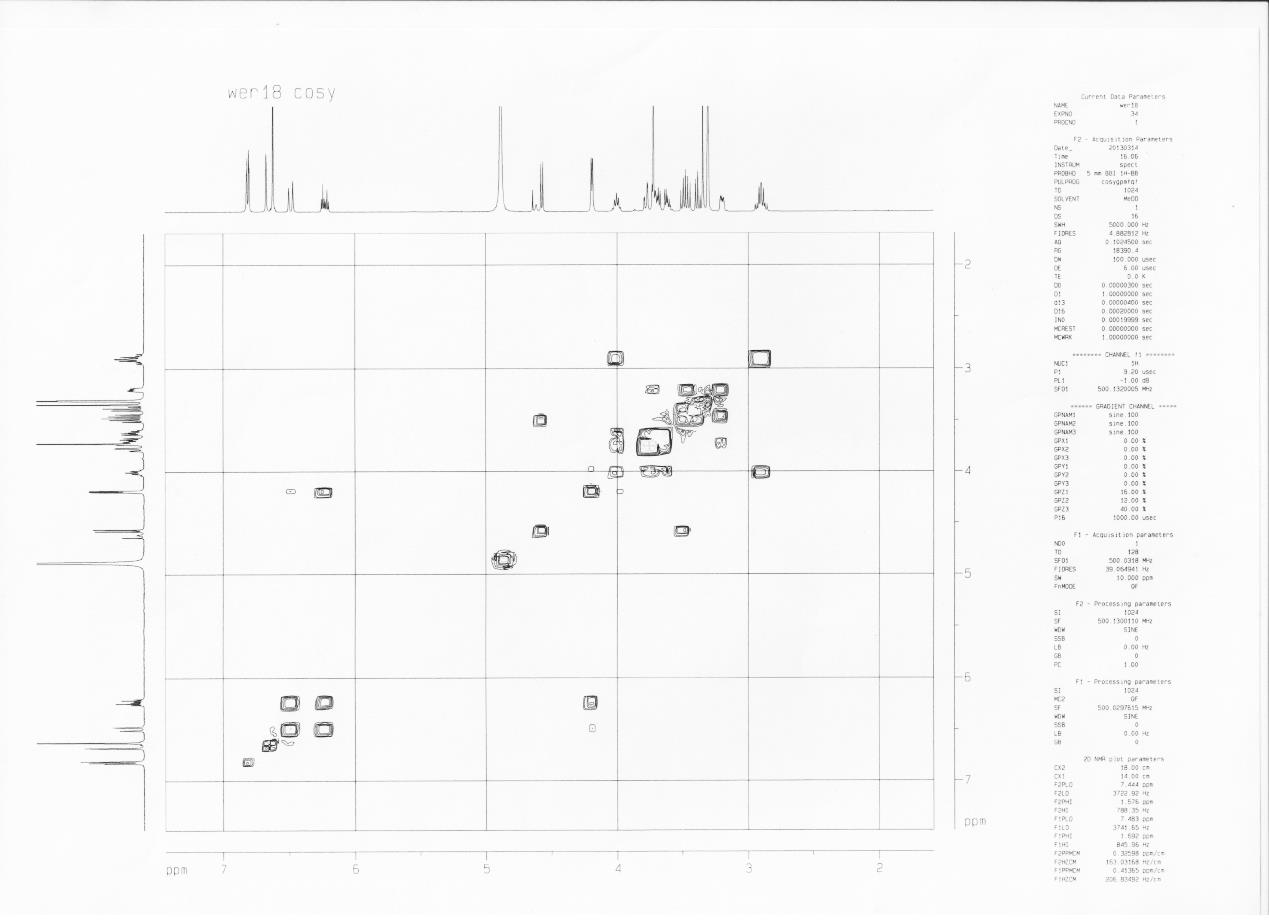


**Figure 14.** COSY spectrum of **2** (400 MHz, methanol-*d*_4_).**
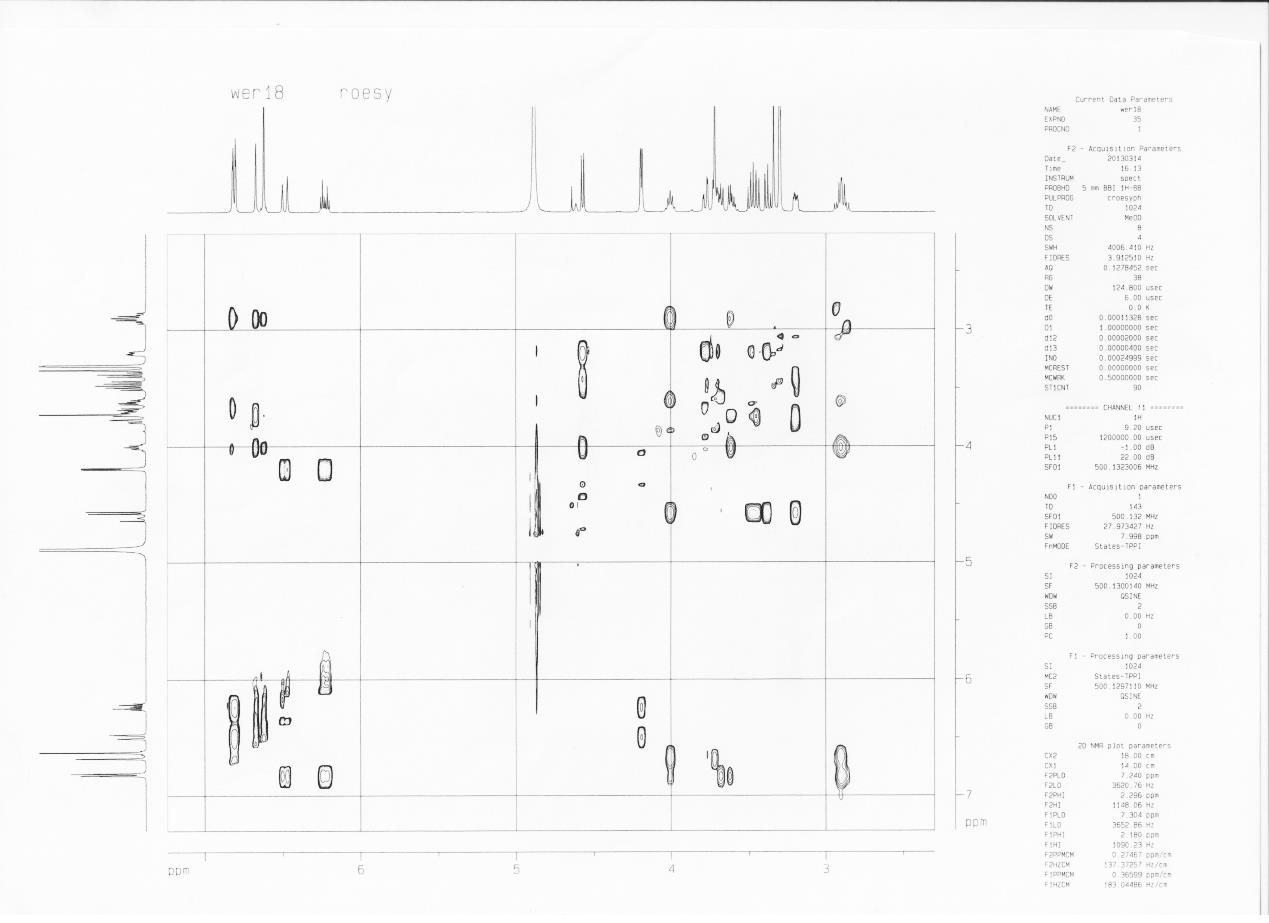
**

**Figure 15.** ^1^H NMR spectrum of **2** (400 MHz, methanol-*d*_4_).


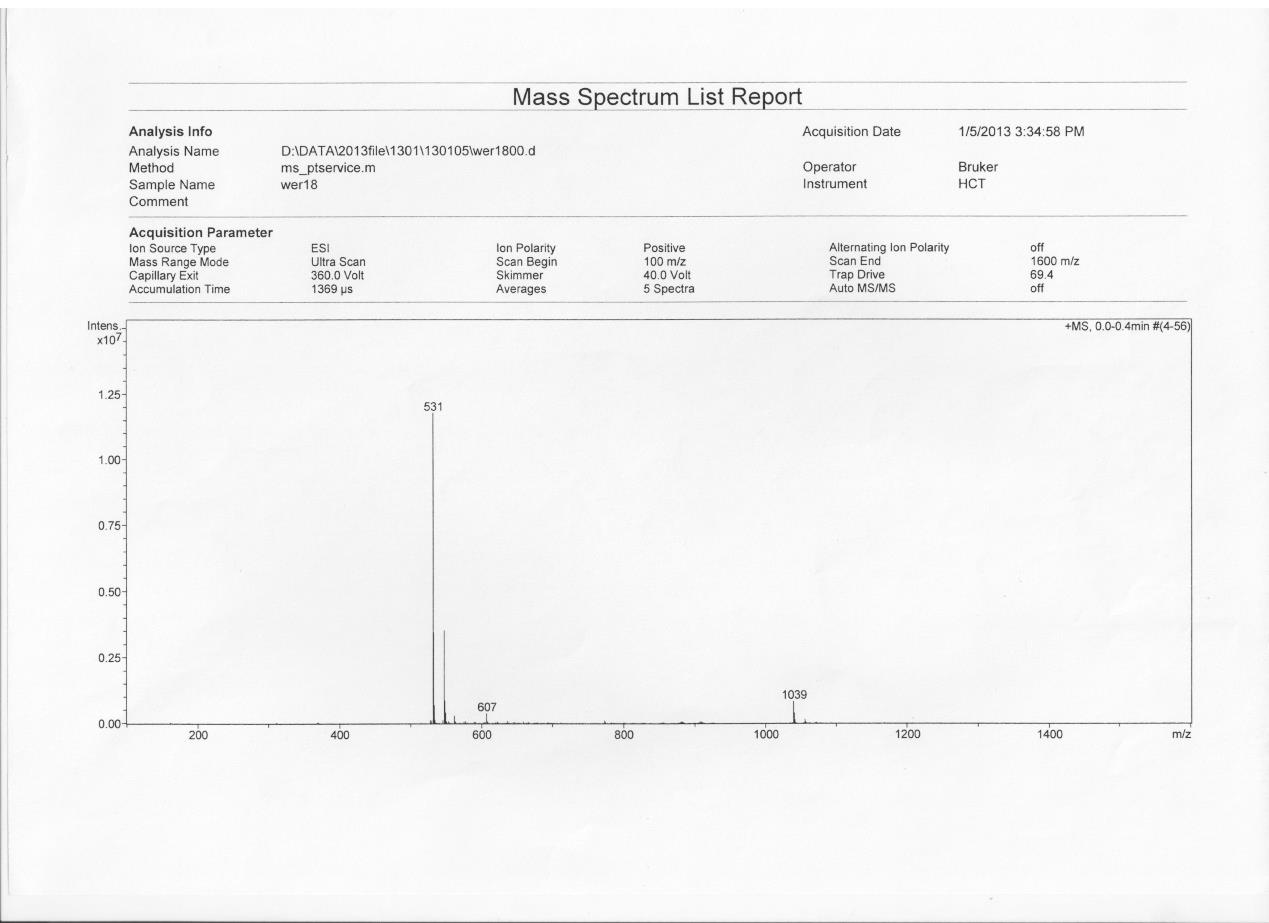


**Figure 16. ESI**-MS spectrum of **2**

**
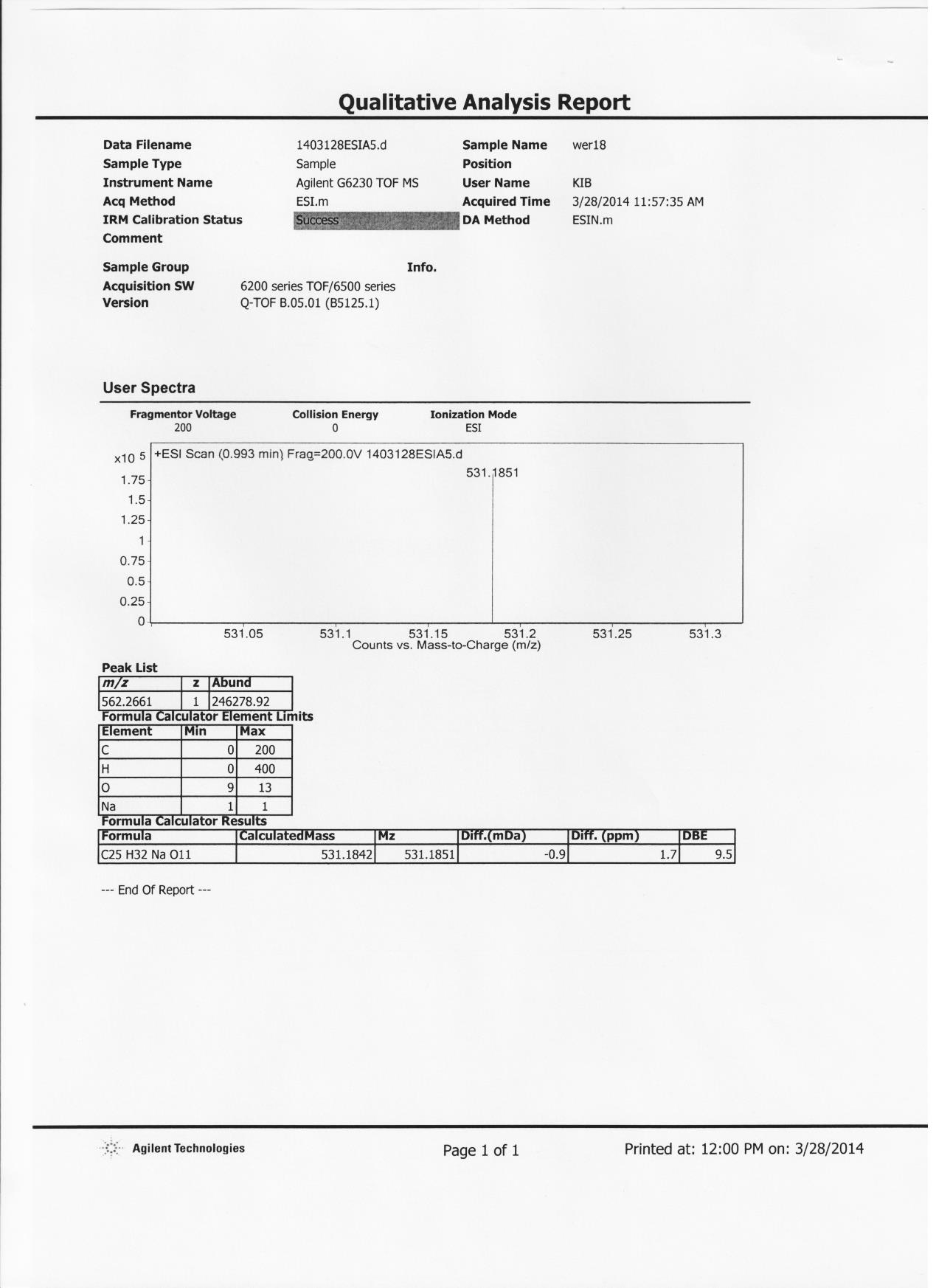
**

**Figure 17. HR**ESI-MS spectrum of **2**


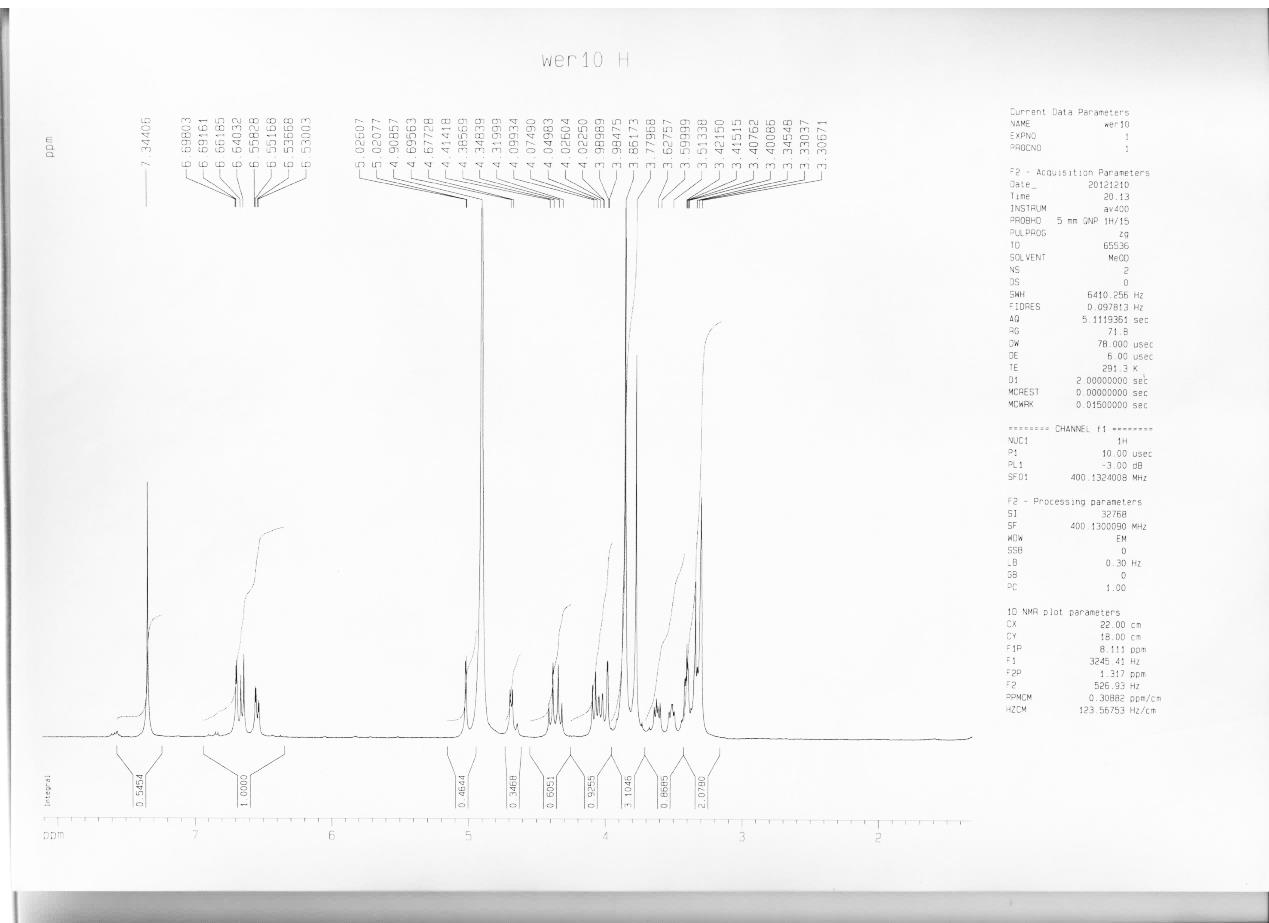


**Figure 18.** ^1^H NMR spectrum of **3** (400 MHz, methanol-*d*_4_).


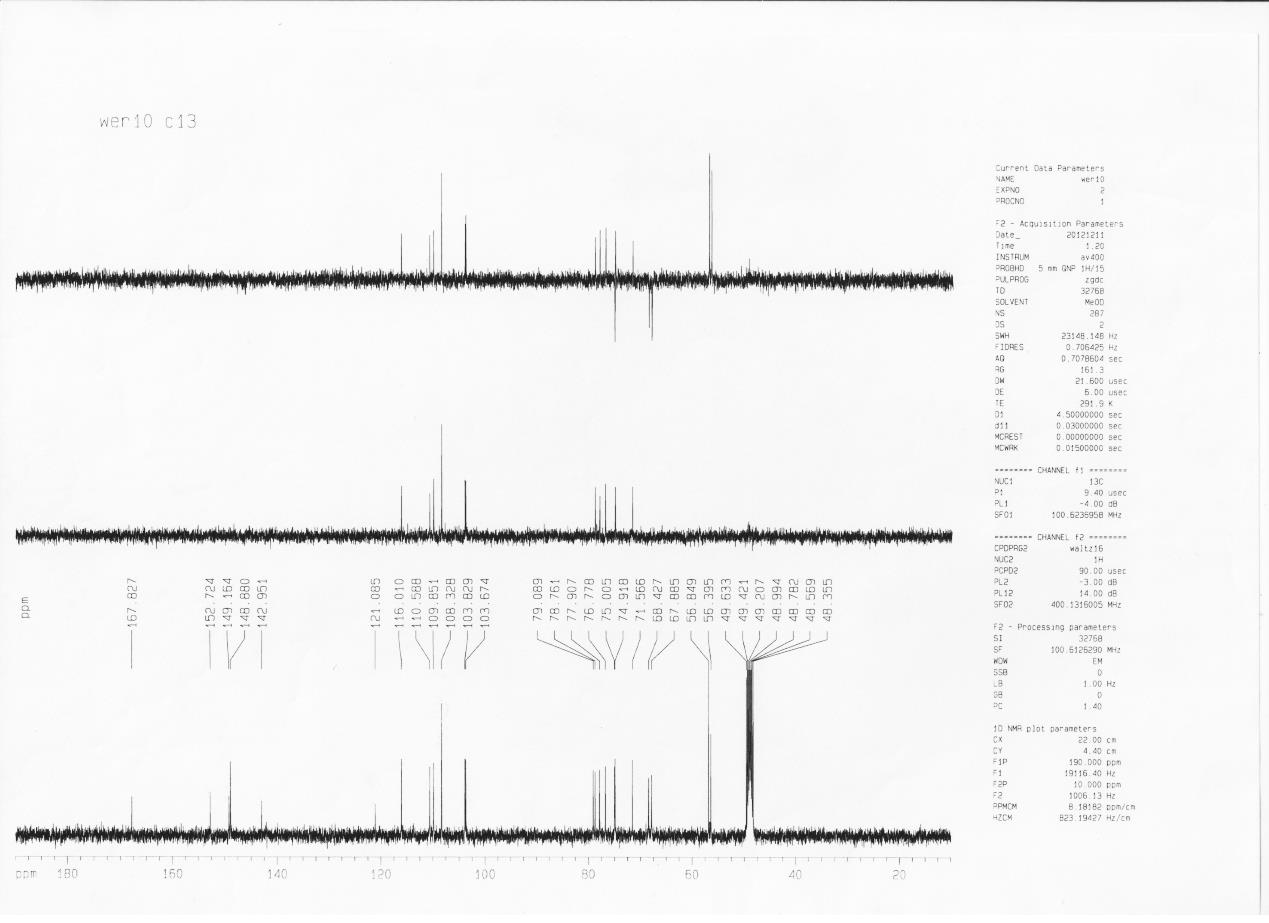


**Figure 19.** ^13^C NMR spectrum of **3** (400 MHz, methanol-*d*_4_).


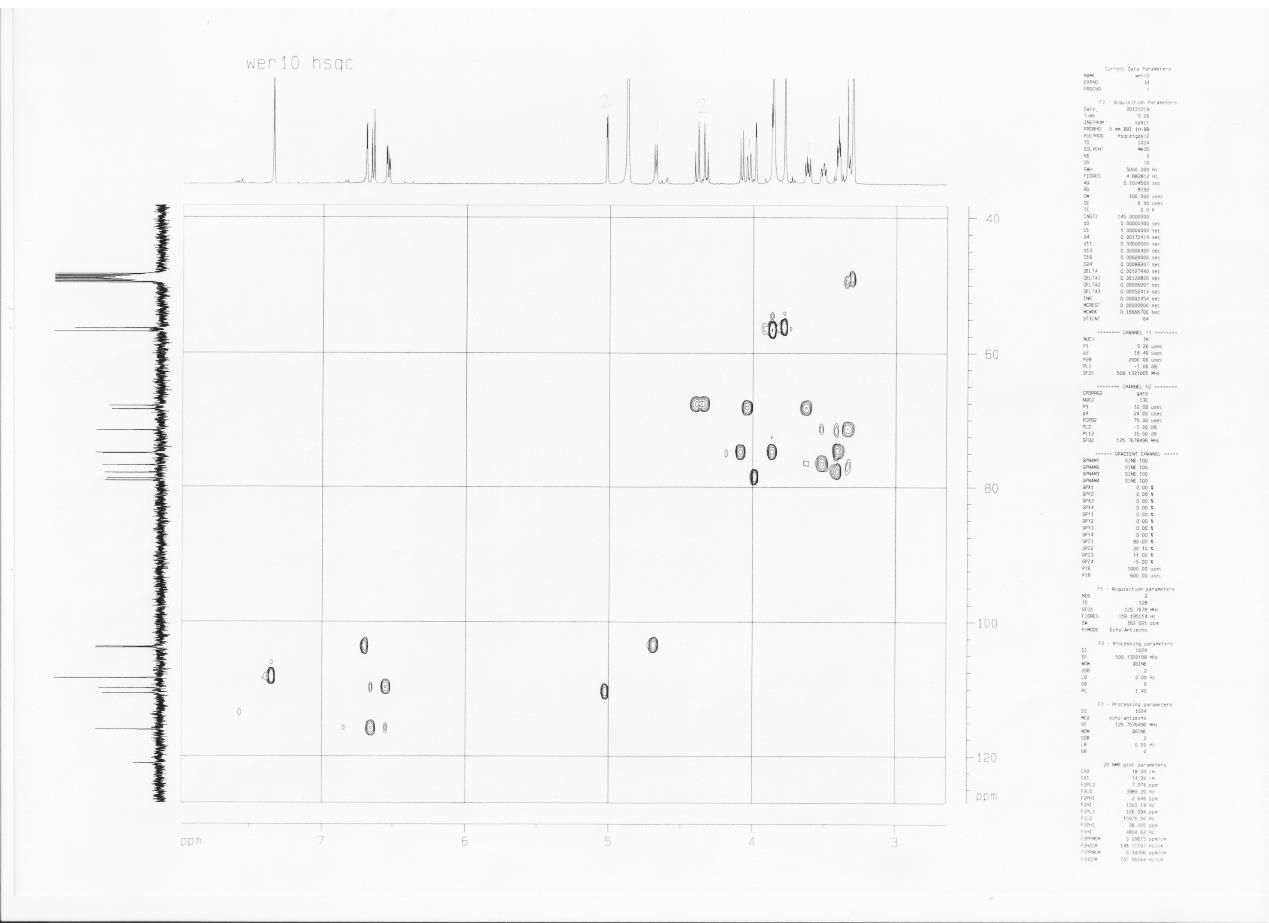


**Figure 20.** HSQC spectrum of **3** (400 MHz, methanol-*d*_4_).


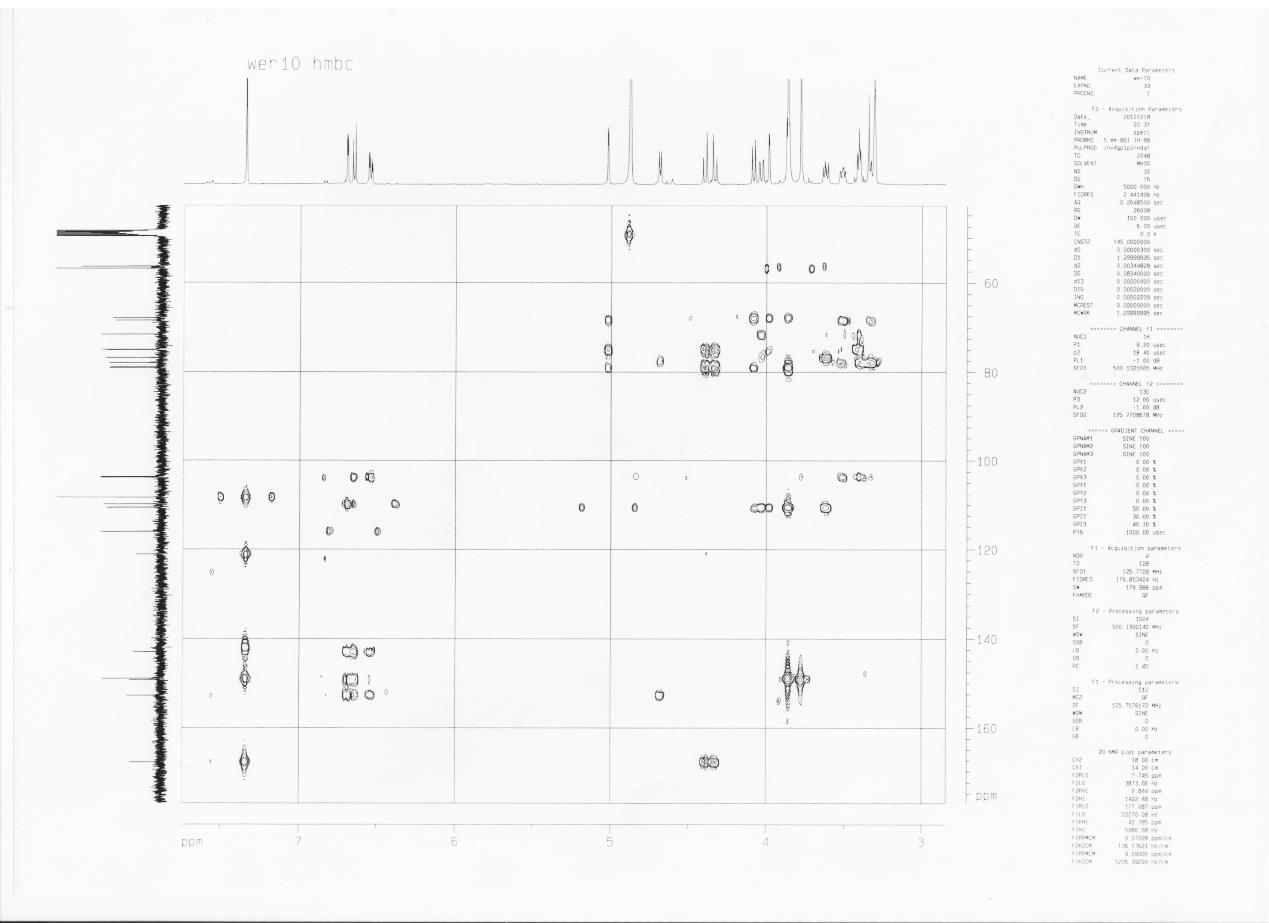


**Figure 21.** HMBC spectrum of **3** (400 MHz, methanol-*d*_4_).


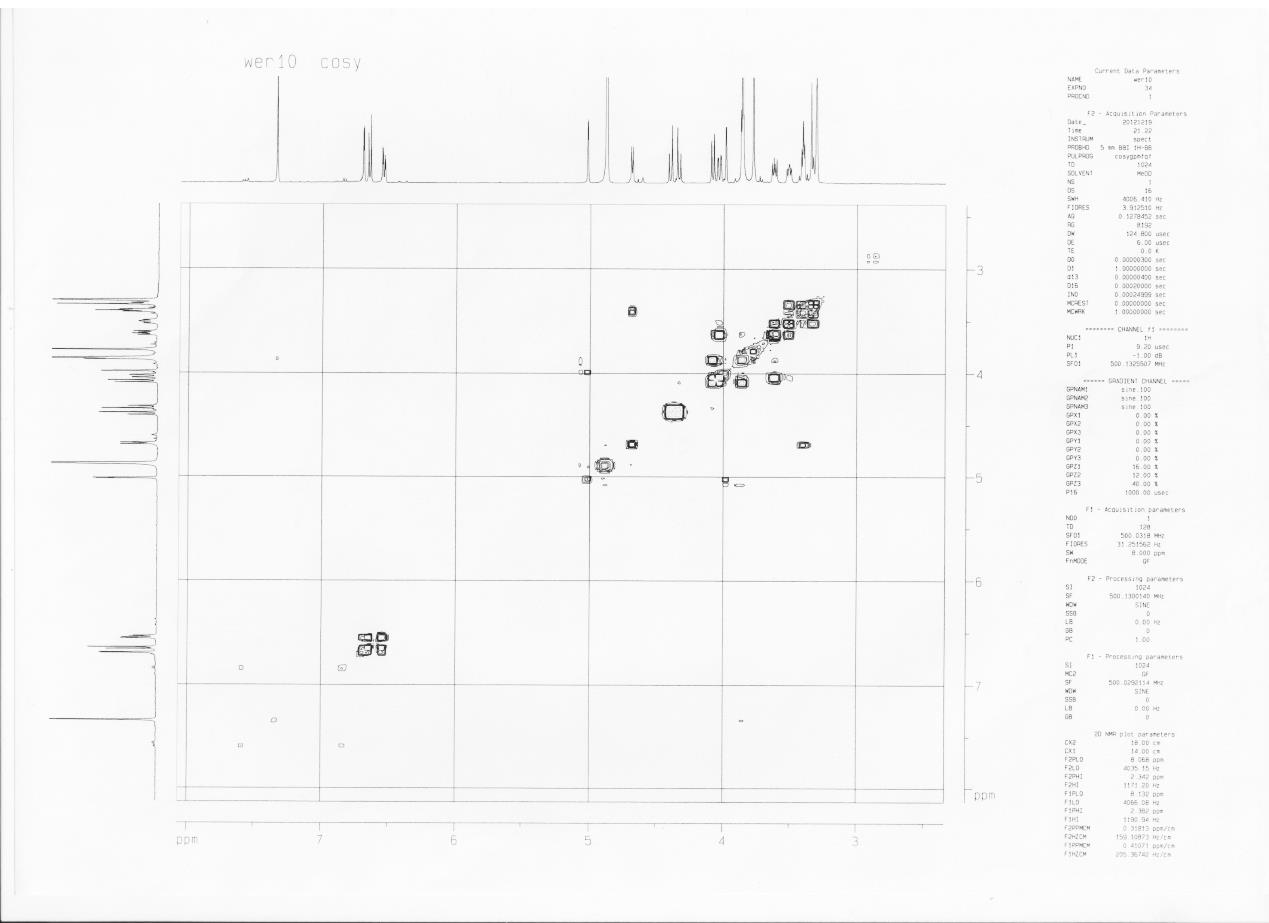


**Figure 22.** COSY spectrum of **3** (400 MHz, methanol-*d*_4_).


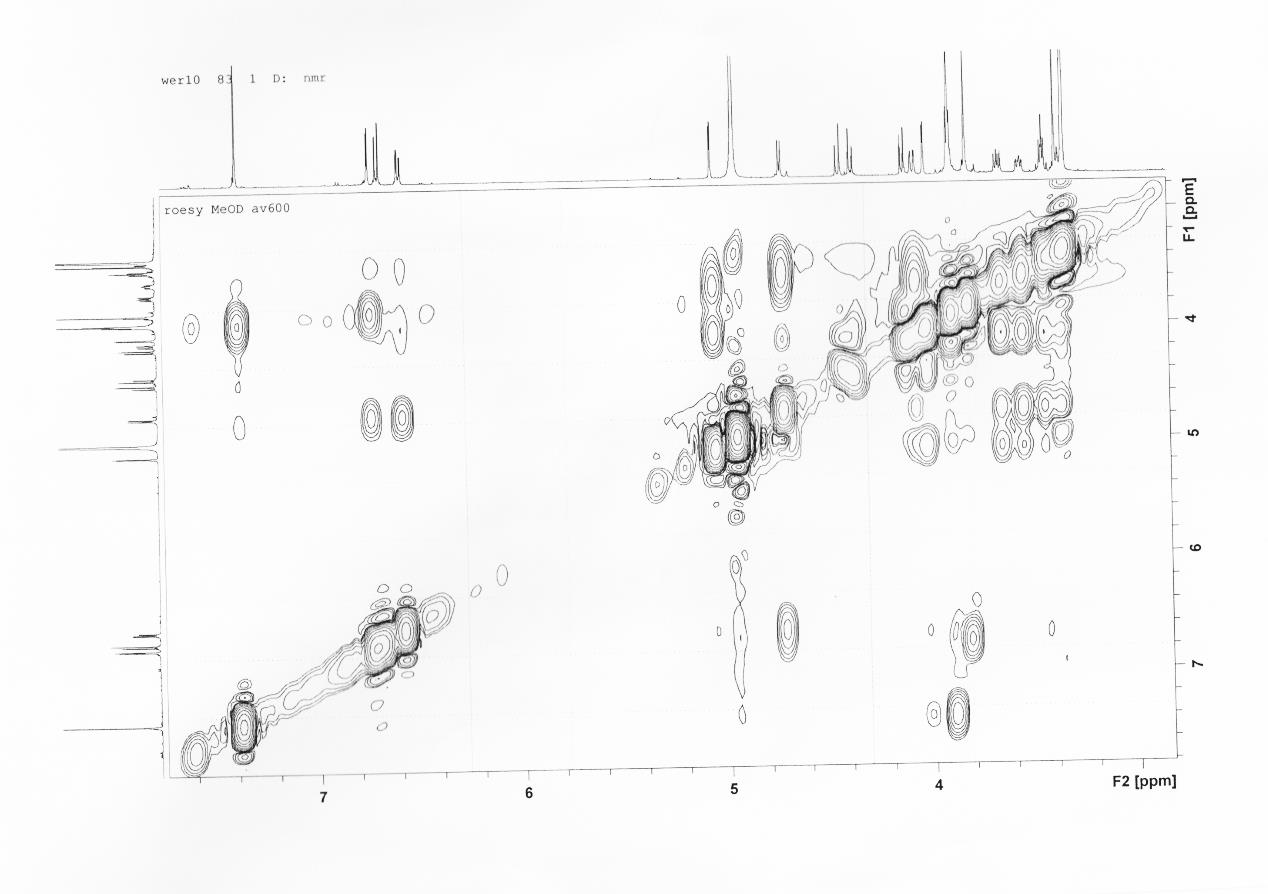


**Figure 23.** ^1^H NMR spectrum of **3** (400 MHz, methanol-*d*_4_).


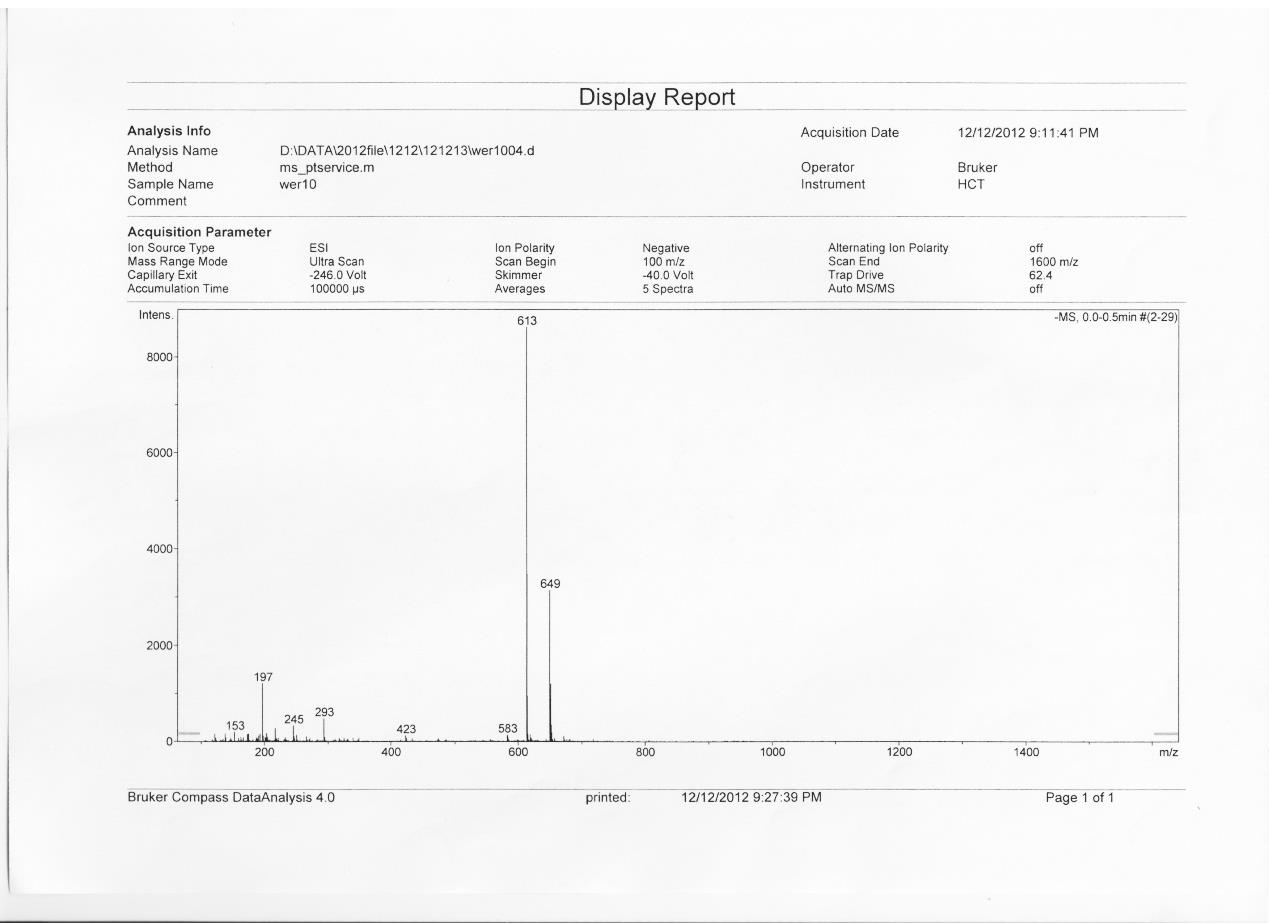


**Figure 24.** ESI-MS spectrum of **3**


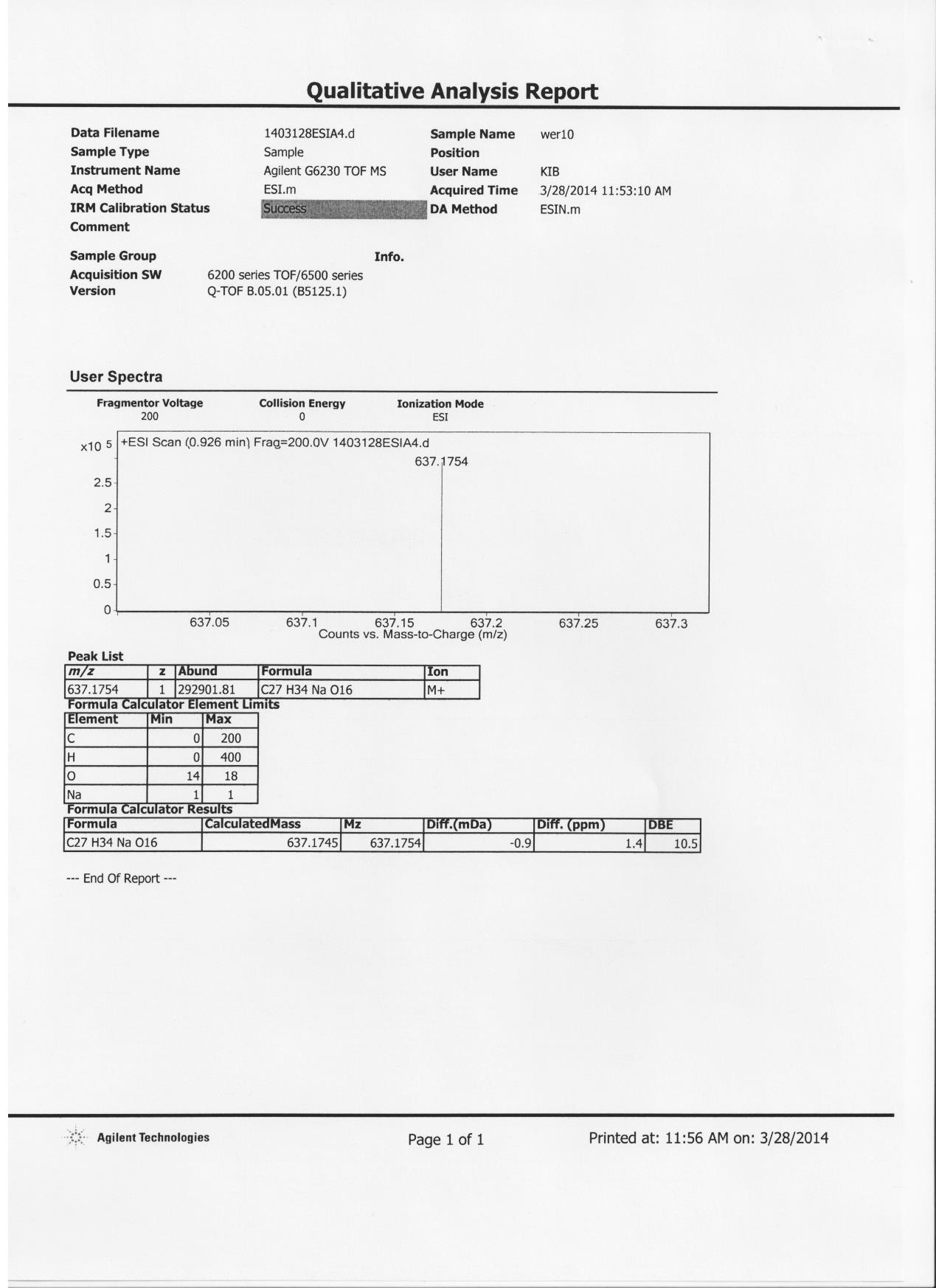


**Figure 25. HR**ESI-MS spectrum of **3**


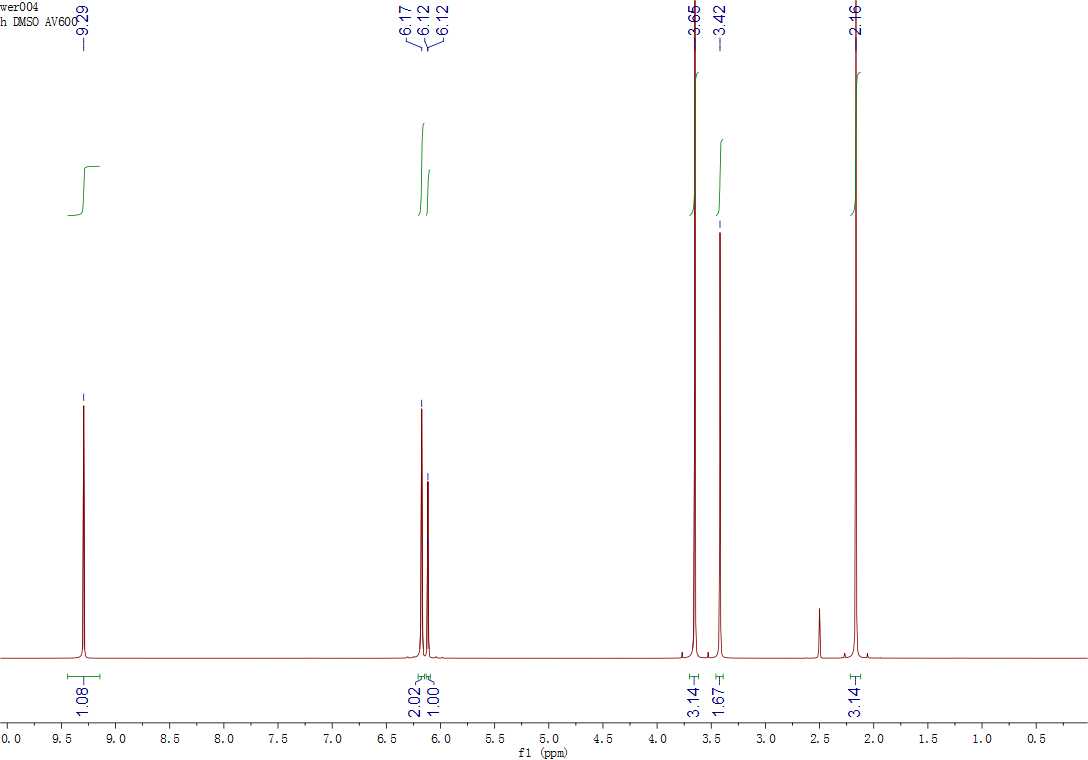


**Figure 26.** ^1^H NMR spectrum of 3-methoxy-5-methylpehnol (600 MHz, dmso-*d*_6_).


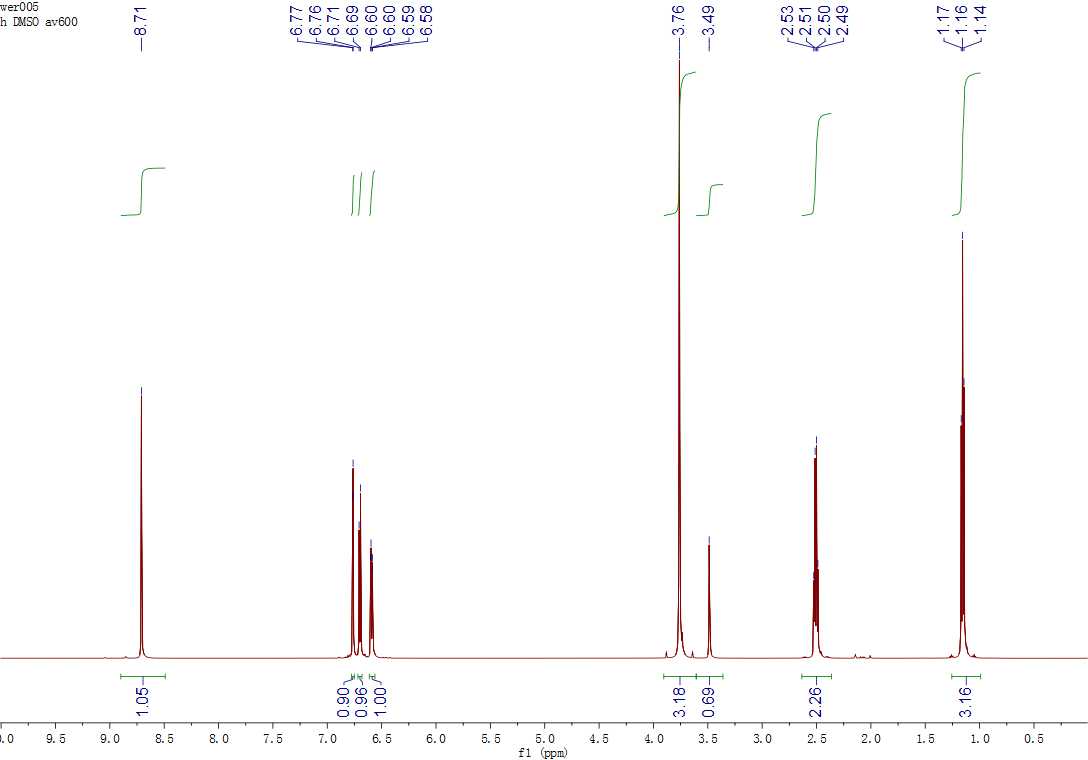


**Figure 27.** ^1^H NMR spectrum of 4-ethyl-2-methoxyphenol (600 MHz, dmso-*d*_6_).
